# Multi-Omics Profiling Identifies Microglial Annexin A2 as a Key Mediator of NF-κB Pro-inflammatory Signaling in Ischemic Reperfusion Injury

**DOI:** 10.1016/j.mcpro.2024.100723

**Published:** 2024-01-20

**Authors:** Xibin Tian, Wuyan Yang, Wei Jiang, Zhen Zhang, Junqiang Liu, Haijun Tu

**Affiliations:** 1 State Key Laboratory of Chemo/Biosensing and Chemometrics, College of Biology, Hunan University, Changsha, Hunan, China; 2 Shenzhen Research Institute, Hunan University, Shenzhen, Guangdong, China

**Keywords:** cerebral ischemia-reperfusion, multi-omics, inflammation, oxygen-glucose deprivation and reoxygenation, microglia, neuronal death

## Abstract

Cerebral stroke is one of the leading causes of mortality and disability worldwide. Restoring the cerebral circulation following a period of occlusion and subsequent tissue oxygenation leads to reperfusion injury. Cerebral ischemic reperfusion (I/R) injury triggers immune and inflammatory responses, apoptosis, neuronal damage, and even death. However, the cellular function and molecular mechanisms underlying cerebral I/R-induced neuronal injury are incompletely understood. By integrating proteomic, phosphoproteomic, and transcriptomic profiling in mouse hippocampi after cerebral I/R, we revealed that the differentially expressed genes and proteins mainly fall into several immune inflammatory response–related pathways. We identified that Annexin 2 (Anxa2) was exclusively upregulated in microglial cells in response to cerebral I/R *in vivo* and oxygen-glucose deprivation and reoxygenation (OGD/R) *in vitro*. RNA-seq analysis revealed a critical role of Anxa2 in the expression of inflammation-related genes in microglia *via* the NF-κB signaling. Mechanistically, microglial Anxa2 is required for nuclear translocation of the p65 subunit of NF-κB and its transcriptional activity upon OGD/R in BV2 microglial cells. Anxa2 knockdown inhibited the OGD/R-induced microglia activation and markedly reduced the expression of pro-inflammatory factors, including TNF-α, IL-1β, and IL-6. Interestingly, conditional medium derived from Anxa2-depleted BV2 cell cultures with OGD/R treatment alleviated neuronal death *in vitro*. Altogether, our findings revealed that microglia Anxa2 plays a critical role in I/R injury by regulating NF-κB inflammatory responses in a non-cell-autonomous manner, which might be a potential target for the neuroprotection against cerebral I/R injury.

## Highlights

• Multi-omics reveals immune-related events in cerebral ischemic reperfusion.
• Anxa2 is exclusively upregulated in microglia in response to OGD/R.
• Anxa2 knockdown reduces inflammatory response and apoptosis induced by OGD/R.
• Anxa2 knockdown inhibits NF-κB signaling activation triggered by OGD/R.
• Microglial Anxa2 knockdown with OGD/R treatment alleviated neuronal death.

## Graphical Abstract

**Figure:**
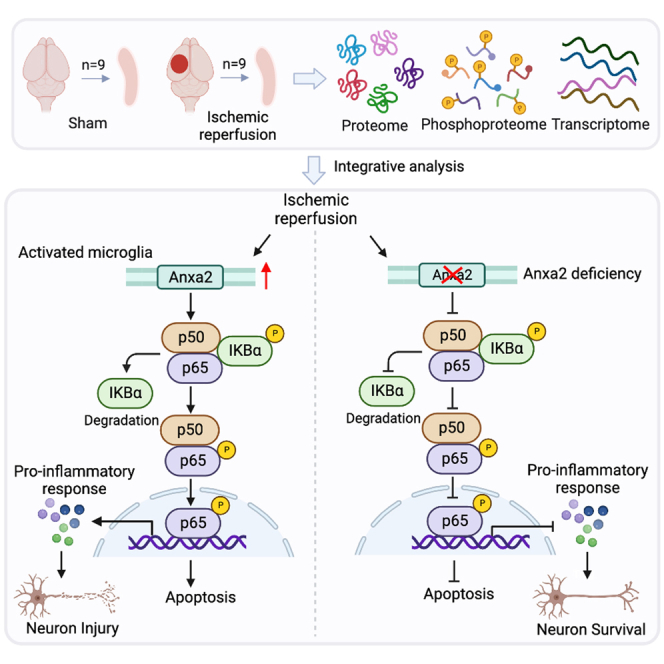

## In Brief

To investigate the cellular function and molecular mechanisms underlying cerebral I/R-induced neuronal injury. we integrated approach to study proteomic, phosphoproteomic, and transcriptomic profiles in mouse hippocampi after cerebral ischemic reperfusion injury. Bioinformatic analysis revealed that upregulated proteins or genes fall into a variety of biological processes of immune inflammatory response. Moreover, functional analysis identifies Annexin 2 as a key molecule in cerebral ischemic reperfusion–triggered inflammatory response.

Cerebral stroke is a devastating and debilitating cerebrovascular disease, that causes high disability and mortality, and has emerged as one of the major public health issues around the world (1, 2, 3). More than 80% of cerebral strokes are ischemic stroke (4, 5, 6). Clinically, ischemic stroke is triggered by a blood vessel in the brain becoming blocked or narrowed, and restoring blood flow quickly is the direct therapeutic treatment for this disease (7). Currently, the only FDA-approved thrombolytic therapy is tissue plasminogen activator (tPA), of which a narrow therapeutic window (<4.5 h) is allowed. Thus, tPA can only be beneficial to a limited percentage of ischemic stroke patients (8, 9). Furthermore, reperfusion after thrombolysis can cause severe secondary damage, aggravating the inflammatory response and risk of hemorrhagic transformation, even disability, or mortality (4, 10). Therefore, it is urgent to find new potential therapeutic targets for cerebral ischemic stroke. Numerous studies have demonstrated that immune and inflammatory response plays a crucial role in brain damage following ischemic reperfusion (11, 12). Therefore, revealing the underlying cellular mechanisms that target immune and inflammatory responses would provide a novel therapeutic target for cerebral ischemia.

Accumulating evidence has concluded that microglia is the resident immune cell in the central nervous system and plays a key role in regulating immune and inflammatory responses after brain damage (13, 14). Microglia are in a “resting” state under physiological conditions and are particularly responsive to changes in the microenvironment of the central nervous system. Specifically, microglial activation is one of the first events that occurs after cerebral ischemia reperfusion (I/R) injury (15, 16). Once activated, microglia are known to exhibit pro- and anti-inflammatory effects. In pro-inflammatory states, activated microglia can produce a number of factors such as tumor necrosis factor-α (TNF-α), interleukin-6 (IL-6), interleukin-1beta (IL-1β), and reactive oxygen species (ROS). They are typically identified by the cell surface markers, including inducible nitric oxide synthase (iNOS), CD16, and CD68 (17, 18). In anti-inflammatory states, activated microglia are characterized by cell surface expression of tumor growth factor-β, arginase-1 (Arg-1), CD206, and interleukin-10 (17, 19). Previous studies have demonstrated that immune and inflammatory response was involved in cerebral I/R injury (20, 21). After cerebral I/R, microglia rapidly migrate toward the lesion site and are further activated. Pro-inflammatory microglia induce inflammatory response, and the production of ROS exacerbates tissue injury, which can stimulate neurotoxicity and eventually lead to neuronal death (13, 22, 23). Therefore, understanding the regulatory function of microglia in immune and inflammatory responses is expected to develop effective therapeutic approaches for cerebral ischemia.

Annexin A2 (Anxa2) is a multifunctional calcium (Ca^2+^) and phospholipid-binding protein that is expressed in a broad spectrum of cells (24). Anxa2 promotes retinal neoangiogenesis in oxygen-induced retinopathy by enhancing perivascular fibrin clearance (25). The hypoxic and ischemic environments in human retinal endothelial cells promote cell autophagy and the survival of retinal endothelial cells *via* the HIF-1/Anxa2 signaling pathway (26). Anxa2 also regulates cerebral endothelial permeability *via* F-actin–VE-cadherin interactions and Robo4-paxillin-ARF6 signaling after cerebrovascular injury (27). The low dose of tPA plus recombinant Anxa2 treatment increases microvessel density, synaptophysin, and VEGF expression in peri-infarct regions, improving long-term neurological outcomes after embolic focal ischemia in rats (28). Anxa2 promotes angiogenesis after ischemic stroke *via* AKT/ERK pathways (29). It also maintains the membrane integrity of late endosomes to modulate inflammasome activation and cytokine secretion in inflammatory dendritic cells (30). Anxa2 binds to endosomes and negatively regulates bacteria-triggered inflammatory responses *via* the TRAM-TRIF pathway (31). Anxa2 regulates ROS and inflammatory response *via* IL-17 signaling in polymicrobial sepsis (32). Anxa2 deficiency exacerbates neutrophil infiltration, neuroinflammation, and long-term neurological outcomes after traumatic brain injury (33). Moreover, recombinant annexin A2 binds to Toll-like receptor 4 in leukocytes and inhibits leukocyte brain infiltration, neuroinflammation, and neuronal cell death after traumatic brain injury (34). However, the cellular and molecular mechanisms underlying the role of Anxa2 in modulating microglial activation in cerebral I/R pathogenesis remain poorly investigated.

Nuclear factor-kappa B (NF-κB) is a transcription factor widely associated with inflammatory responses following ischemia and other neuroinflammatory disorders (23). Genetic and pharmacological studies targeting NF-κB-activating IKK showed that inhibiting NF-κB generally benefits stroke recovery (35, 36). Activated microglia can enable different inflammatory pathways, including the NF-κB signaling pathway. It can trigger the release of many pro-inflammatory mediators, such as TNF-α, IL-1β, and IL-6, which lead to inflammatory reactions. The production of inflammatory factors can aggravate the damage of neighboring neurons and result in tissue injury. Therefore, we examined whether Anxa2 regulates microglial activation and targets these signaling molecules or pathways after cerebral I/R.

In this study, we performed combinatorial analyses of proteome, phosphoproteome, and transcriptome and identified Anxa2 as a crucial molecule for regulating the immune-inflammatory response in microglia. Loss of Anxa2 expression impairs gene expression signatures associated with inflammation in microglia. Anxa2 is required for microglial activation and the production of pro-inflammatory factors *via* the NF-κB signaling pathway by facilitating the nuclear translocation of the p65 subunit. Importantly, loss of Anxa2 attenuates neuronal death in response to oxygen-glucose deprivation and reoxygenation (OGD/R) in a non-cell-autonomous manner. Thus, our findings reveal a critical role of microglial Anxa2 in regulating the inflammatory response, suggesting that Anxa2 may be a potential therapeutic target for cerebral ischemia.

## Experimental Procedures

### Animal

All animal experiments were performed with approval from the Hunan University Animal Ethics Committee (No. SYXK [Xiang] 2023-0010). In this study, adult C57BL/6J male mice (8 weeks old) weighing 22 to 26 g were randomly divided into the sham or I/R groups. Mice were kept in standard conditions, with sterile cages, bedding, water, and food, with 12 h of light and 12 h of dark cycle.

### Cerebral Ischemia-Reperfusion Model

The cerebral I/R model was induced by extracranial intraluminal middle cerebral artery occlusion (MCAO), as previously described (37). Briefly, 8 weeks old mice were anesthetized with 5% isoflurane and maintained with 1% isoflurane (RWD Life Science, R511-22) in an oxygen/air mixture by using a gas anesthesia mask (RWD Life Science, R580SRWD). Under the operating stereo microscope, the left common carotid artery, the external carotid artery (ECA), and the internal carotid artery were sequentially exposed. The ECA was ligated with a 5–0 silk suture, and a 2 cm length of silicon-rubber-coated monofilament (RWD Life Science, MSMC24B104PK50) was inserted from the ECA through the internal carotid artery up to the bifurcation of the left middle cerebral artery and anterior cerebral artery to block the circulation in the left middle cerebral artery territory. After 90 min of occlusion, blood flow was restored by the withdrawal of the inserted filament (Reperfusion). The sham-operated mice underwent the same experimental procedures except for the filament insertion. Cerebral blood flow was monitored using a Laser Speckle Imaging System (RWD Life Science, RFLSI III) during MCAO and reperfusion. Mice were sacrificed 24 h post-reperfusion.

### Experimental Design and Statistical Rationale

For proteomic and transcriptomic analyses, nine hippocampi from the sham or MCAO/R group were analyzed by three biological replicate MS runs (3 hippocampi were pooled per MS run). Five percent (40 μg) of the pooled samples were used for whole proteomic analysis, and the remaining 95% (760 μg) were subjected to phosphoproteomic profiling. For BV2 cell transcriptomic analysis, three biological replicates were used to compare genes in each stable knockdown cell line. Each biological replicate comprises three technical replicates (1 μg of total RNA). For all omics analyses, the differentially expressed genes (DEGs)/proteins (DEPs, *p*-value <0.05; fold-change <0.8 [downregulated] or fold-change >1.2 [upregulated]) were subjected to Kyoto Encyclopedia of Genes and Genomes (KEGG) pathway analysis.

### Protein Extraction, Trypsin Digestion, and TMT Labeling

Protein extraction, trypsin digestion, and tandem mass tag (TMT) labeling were performed as described previously (38). After cerebral I/R, hippocampi from the ipsilateral hemispheres were grounded in liquid nitrogen and then resuspended with four volumes of lysis buffer (8 M urea, 1% protease inhibitor mix). The mixture was sonicated three times on ice with a high-intensity ultrasonic processor. Tissue debris was removed by centrifugation at 12,000*g* at 4 °C for 10 min. Finally, the supernatant was collected, and the protein concentration was determined with a BCA kit according to the manufacturer’s instructions.

For digestion, the protein solution was reduced with 5 mM DTT for 30 min at 56 °C and alkylated with 11 mM iodoacetamide for 15 min at room temperature in the dark. Then, 100 mM TEAB was added to decrease the urea concentration below 2 M. Finally, trypsin was added at a 1:50 trypsin-to-protein mass ratio for the first digestion overnight and at a 1:100 ratio for the second digestion for 4 h.

For TMT labeling, peptides were desalted with a Strata X C18 SPE column (Phenomenex) and vacuum dried. They were then reconstituted in 0.5 M TEAB and processed according to the manufacturer’s protocol. Briefly, one unit of TMT reagent was thawed and reconstituted in acetonitrile. The peptide mixtures were then incubated for 2 h at room temperature and pooled, desalted, and dried by vacuum centrifugation.

### Phosphopeptide Enrichment

Phosphopeptide enrichment was performed following our previously published method (38). Phosphopeptide mixtures were suspended using immobilized metal affinity chromatography (IMAC) microsphere suspensions with vibration in loading buffer (50% acetonitrile/6% TFA). The IMAC microspheres were collected by centrifugation, washed with 50% acetonitrile/6% trifluoroacetic acid, and again with 30% acetonitrile/0.1% trifluoroacetic acid. The enriched phosphopeptides were eluted with 10% ammonium hydroxide from the IMAC microspheres and analyzed with liquid chromatography-tandem mass spectrometry (LC–MS/MS).

### Liquid Chromatography-Mass Spectrometry Analysis and Database Search

LC-MS/MS analysis and data search were performed according to our previous methods (38). The peptides were dissolved in 0.1% formic acid (solvent A) and loaded onto a homemade reversed-phase analytical column (15 cm length, 75 μm i.d.). The gradient was comprised of an increase from 6% to 23% solvent B (0.1% formic acid in 98% acetonitrile) over 26 min, 23% to 35% in 8 min, and climbing to 80% in 3 min, then holding at 80% for the last 3 min, all at a constant flow rate of 400 nl/min on an EASY-nLC 1000 UPLC system. The peptides were subjected to an NSI source followed by tandem mass spectrometry (MS/MS) in a Q Exactive TM Plus (Thermo) coupled online to UPLC. The electrospray voltage of 2.0 kV was applied, the m/z scan range from 350 to 1800 was used for the full scan, and intact peptides were detected in the Orbitrap at a resolution of 70,000. Peptides were then selected for MS/MS using the NCE setting of 28, and the fragments were detected in the Orbitrap at a resolution of 17,500. A data-dependent procedure was alternated with one MS scan followed by 20 MS/MS scans with 15.0 s dynamic exclusion. Automatic gain control was set at 50,000, and the fixed first mass was set at 100 m/z. The resulting MS/MS data were processed using the MaxQuant search engine (v.1.5.2.8). Tandem mass spectra were searched against the SwissProt Mouse database concatenated with the reverse decoy database (released February 2018; 16,964 sequences). Carbamidomethyl on cysteines was specified as a fixed modification. Acetylation on the N-terminal of proteins and oxidation on methionine were set as variable modifications for the proteomic analysis. N-terminal protein acetylation, oxidation on methionine, and phosphorylation on serine, threonine, and tyrosine were specified as variable modifications for the phosphoproteomic analysis. Trypsin/P was specified as a cleavage enzyme, allowing up to four missing cleavages. The mass tolerance for precursor ions was set as 20 ppm in the first search and 5 ppm in the main search, and the mass tolerance for fragment ions was set as 0.02 Da. FDR was adjusted to <1% and the minimum score for peptides or modified peptides was set to >40.

### Transcriptome Sequencing

After cerebral I/R, ipsilateral hippocampi were dissected rapidly, washed with PBS, immediately snap-frozen in liquid nitrogen, and stored at −80 °C until use. Total RNA was extracted using Trizol reagent (Invitrogen) according to the manufacturer’s protocol. cDNA library generation and RNA sequencing were performed by NovoGene, and the details were described previously (39). The clean data was aligned by Tophat 2 to the reference genome of the species which was derived from the Ensembl database (http://www.ensembl.org/) and annotated according to the collected information from UniProtKB, Ensembl, gene ontology (GO), KEGG, and eggNOG. Finally, based on the number of sequences aligned to the gene, the expression level was calculated using HTSeq 0.6.1p2.

### Combinatorial Analyses of Transcriptome, Proteome, and Phosphoproteome

After data normalization, DEGs and DEPs between I/R *versus* sham groups were used for subsequent bioinformatical analyses. The GO and KEGG enrichment analyses were performed with the ‘clusterProfiler’ package (v. 4.4.4) with a *p*-value threshold set to 0.05. The hierarchical cluster analysis was conducted by the ‘pheatmap’ package (v. 1.0.8) in RStudio (v. 4.2.1). The principal component analysis (PCA) analysis was performed using TBtools (v. 1.108). For gene network analysis, DEGs were input into STRING (https://string-db.org/) database to obtain the inner interaction, and the gene-gene network was visualized by Cytoscape software (v. 3.9.1). CytoHubba plug-in (v. 0.1) was used to predict the important gene and subnetwork based on the topological algorithms of Maximal Clique Centrality.

### Generation of Anxa2 Knockdown Stable Cell Lines

HEK293T cells and BV2 microglial cells were maintained in Dulbecco’s modified Eagle’s medium (DMEM; Corning) supplemented with 10% fetal bovine serum (FBS; Gibco) and 1% penicillin-streptomycin antibiotics (Thermo Fisher Scientific) in a humidified 5% CO_2_ incubator at 37 °C.

To generate Anxa2 knockdown cell lines, lentiviral particles were prepared according to a previously described protocol (40). HEK293T cells were cotransfected with either Anxa2-targeting shRNA (Sigma, TRCN00000110695) or a control shRNA expressed from the pLKO.1 vector (Addgene, 10878) with the pSPAX2 packaging plasmid (Addgene, 12260) and the pMD2.G envelope plasmid (Addgene, 12259) using Lipofectamine 2000 (Thermo Fisher Scientific, 11668019) in Opti-MEM medium. Tissue culture supernatants were collected 72 h after transfection and filtered through a 0.45 μm PVDF membrane filter. The harvested lentivirus was aliquoted and stored at −80 °C for later use.

For all the lentivirus infections, BV2 cells were plated at 2 × 10^4^ cells per well in 6-well plates overnight, and the cells were transduced with virus-containing medium for 72 h. Subsequently, cells were treated with puromycin (Invivogen, ant-pr-1) for 48 h at a final concentration of 1 μg/ml. After puromycin selection, stable cell lines were obtained and maintained in the standard complete medium. Cells were further treated with OGD/R or other conditions as indicated. The stable Anxa2 knockdown BV2 line and the control cells were called shAnxa2 and shCtrl, respectively. The generation of GFP and Anxa2-GFP stable cell lines followed the abovementioned method.

### Mouse Primary Neuron, Microglia, and Astrocyte Cultures

Mouse primary neuronal cultures were prepared as described previously (41) with minor modifications. Briefly, the hippocampi were dissected from newborn mice, carefully stripped of their meninges. They were then digested with 0.25% trypsin for 10 min at 37 °C before plating medium (DMEM +10% FBS) was added to stop the enzymatic activity. The tissue was gently triturated through polished glass pipette several times, and the cell suspension was passed through a 70 μm cell strainer (BD Falcon). The single cell suspension was then plated on poly-D-Lysine (Sigma, P6407) precoated coverslips in 24 or 6-well culture plates in Neurobasal medium supplemented with B-27 supplement (Thermo Fisher Scientific, 17504044) and grown at 37 °C in a humiliated 5% CO2 incubator. Half of the medium was replaced every 3 days. Neurons were cultured for 14 days.

Primary microglia cells were cultured as described previously (42) with minor modifications. Briefly, the cortices and hippocampi were removed from postnatal day 1 or 2 (P1 or P2) mice, respectively, and placed in a 60-mm dish with 1 × PBS on ice. Tissues were stripped off the meninges and choroid plexus membranes and then minced with a sterile scissor. They were then digested with 0.25% trypsin for 10 min at 37 °C. After trituration, cell suspensions were passed through a 40 μm cell strainer and centrifuged at 500*g* for 5 min. The cell pellets were resuspended in culture medium (DMEM +10% FBS +1% penicillin/streptomycin) and seeded into poly-D-lysine–coated T75 tissue culture flasks (3 brains/flask) and grown at 37 °C in a humidified 5% CO_2_ incubator. The medium was replaced every 4 days with culture medium supplemented with 5 ng/ml macrophage colony-stimulating factor (Shenandoah Biotechnology). Microglia were collected on day 12 by shaking the flasks at 180 rpm at 37 °C for 4 to 5 h. The floating cells in the supernatant were counted and centrifuged at 500*g* for 10 min. They were then resuspended in a culture medium and plated on poly-D-lysine–coated 12-well plates at a density of 1 × 10^5^ cells per well. After seeding, the microglia were allowed to attach to the plate for 1 h. The plating medium was then removed and rinsed once with the prewarmed culture medium to remove nonadherent cells. The remaining cells in the flask were treated with 0.25% trypsin to obtain primary astrocytes.

### Oxygen-Glucose Deprivation and Reoxygenation Model

The OGD/R model has been described previously (43). BV2 cells were plated in an anaerobic incubator with 5% CO_2_ and 95% atmospheric air overnight. Then, the cells were washed two times with 1 × PBS, incubated with glucose-free DMEM (Gibco, 11966-025), and grown in a hypoxic incubator chamber (94% N_2_, 1% O_2_, and 5% CO_2_) for 4 h (OGD). Following OGD treatment, the medium was replaced with high-glucose DMEM (normal medium), and the cells were returned to a normoxic incubator for 24 h to mimic *in vivo* reperfusion-like conditions. For primary microglia and astrocytes, OGD was performed for 1.5 h instead. To assess the viability of primary neurons following OGD/R, they were subjected to OGD for 1.5 h and were incubated with a maintenance medium containing 50% of BV2 reoxygenation-conditioned medium (BV2-CM) during the 24 h of reoxygenation period (44).

### Immunoblotting and Antibodies

Western blotting was performed as previously described (45). Cells were collected and lyzed in RIPA buffer containing 50 mM Tris, pH 8.0, 150 mM NaCl, 1% TritonX-100, 1% sodium deoxycholate, 0.1% SDS, and 2 mM EDTA supplemented with 1 mM PMSF, protease (Sigma, P8340), and phosphatase (Roche, 04906837001) inhibitor cocktail on ice. After centrifugation at 20,000*g* for 20 min at 4 °C, the supernatants were collected for BCA assay to determine their protein concentrations. Samples were mixed with 5 × SDS sample buffer (0.5 M Tris–HCl pH 6.8, 500 mM NaCl, 5% sodium deoxycholate, 10% SDS, 5% 2-mercaptoethanol, and 60 mM EDTA, 0.1% bromophenol blue) and boiled for 10 min before SDS-PAGE analysis. After electrophoresis, proteins were transferred to nitrocellulose membranes and probed with the indicated antibodies. Actin or GAPDH were used as loading controls.

Primary antibodies for immunoblotting are as follows: anti-Annexin A2 (Santa Cruz Biotechnology, sc-28385); anti-iNOS (Proteintech, 22226-1-AP); anti-Arginase-1 (Proteintech, 16001-1-AP); anti-NF-κB p65 (Cell signaling Technology, 8242); anti-phospho-p65-Ser536 (Cell signaling Technology, 3033); anti-IκBα (Cell signaling Technology, 4814); anti-phospho-IκBα-Ser32 (Cell signaling Technology, 2859); anti-Caspase-3 (Cell signaling Technology, 14,220); anti-cleaved Caspase-3 (Asp175) (Cell signaling Technology, 28599664); anti-Bax antibody (Cell signaling Technology, 14796); anti-Bcl-2 (Cell signaling Technology, 3498); anti-GAPDH (Santa Cruz Biotechnology, sc-365062); anti-β-actin (Santa Cruz Biotechnology, sc-8432); anti-GFAP (Santa Cruz Biotechnology, sc-33673); anti-Tuj1 (GeneTex, GTX130245); anti-Iba1 (Abcam, ab107159).

### Enzyme-Linked Immunosorbent Assay

BV2 microglial cells were seeded in 96-well plates at the density of 2 × 10^4^ cells/well. After OGD/R, the supernatants were collected for TNF-α, IL-1β, and IL-6 detection using commercial ELISA kits (Proteintech) according to the manufacturer’s instructions. The absorbance of the samples was measured at 450 nm using a microplate reader (46).

### Immunoprecipitation

Immunoprecipitation was performed as previously described (47). BV2 cells were grown in 10 cm dishes. After OGD/R, cells were extracted in cold lysis buffer (50 mM Tris–HCl pH 7.5, 150 mM NaCl, 1% Nonidet P-40, 1 mM EDTA, and 0.5% sodium deoxycholate) containing protease inhibitor mixture (Sigma, P8340). Cell lysates were sonicated and centrifuged at 20,000*g* for 10 min at 4 °C. Cleared lysates were incubated with anti-Anxa2 (Proteintech, 11256-1-AP) or anti-p50 (Cell signaling Technology, 13586) antibodies overnight at 4 °C and then precipitated with protein A/G magnetic beads at 4 °C for 2 h. After incubation, the beads were washed three times with washing buffer (50 mM Tris–HCl pH 7.5, 150 mM NaCl, 0.5% Nonidet P-40, 1 mM EDTA, and 0.5% sodium deoxycholate) and eluted with elution buffer (Thermo Fisher Scientific, 21004) at room temperature for 10 min. The eluents were neutralized with 1 M Tris–HCl (pH 8.0) and analyzed by immunoblotting.

### Quantitative Real Time-PCR

Total RNA was extracted from the mouse ipsilateral hemisphere brain and cells using the TRIzol reagent (Life Technologies, 15596018) according to the protocol provided by the manufacturer. RNA was dissolved in diethylpyrocarbonate-treated water and reverse transcribed using the Verso cDNA synthesis kit (Thermo Fisher Scientific, AB-1453) according to the manufacturer’s instructions. cDNA was quantitated with real-time PCR using the SYBR Green Master Mix (Thermo Fisher Scientific, A25742) on a Bio-Rad CFX96 real-time PCR machine. Relative fold changes were calculated using the comparative C_T_ (2^-ΔΔCT^) method and normalized to the *Gapdh* gene. All experiments were performed at least three times. Primer sequences for RT-PCR are shown in supplemental Table S11.

### Subcellular Fractionation Assay

Subcellular fractionations were performed as described previously (48). Cells (2 × 10^6^ cells per sample) were collected, washed with ice-cold PBS, and lyzed in 200 μl of buffer A (20 mM Tris pH 7.6, 0.1 mM EDTA, 2 mM MgCl_2_, 0.5 mM NaF, and 0.5 mM Na_3_VO_4_ supplemented with 1 mM PMSF, 1 × protease inhibitor cocktail) for 15 min in a 1.5 ml tube on ice. The cells were vortexed for 15 s every 5 min. Nonidet P-40 was added at a final concentration of 1% (v/v), and the suspensions were homogenized by vortexing for 10 s. They were then centrifuged for 5 min at 500*g* at 4 °C. This supernatant is the cytoplasmic fraction, while the pellet contains the nuclear fraction. The nuclear pellet was resuspended in 50 μl of extraction buffer B (20 mM Hepes pH 7.9, 400 mM NaCl, 25% (v/v) glycerol, 1 mM EDTA, 0.5 mM NaF, 0.5 mM Na_3_VO_4_, and 0.5 mM DTT) supplemented with 1 mM PMSF, 1 × protease inhibitor cocktail for 30 min on ice with vortexing at 10 min intervals, and then centrifuged for 20 min at 20,000*g* at 4 °C to obtain the cleared nuclear fraction.

### Luciferase Assay

Luciferase assay was performed as described previously (49). BV2 cells were seeded in 24-well plates and cotransfected with pNF-κB-Luc (Addgene, 49343) and Renilla luciferase reporter plasmids (Promega, E2231) using Lipofectamine 2000 for 24 h followed by OGD/R. The luciferase activities were determined using a Dual-Luciferase Reporter Assay kit (Promega, E1910) according to the manufacturer’s instructions.

### Cell Viability Assay

The CCK8 assay was used to assess BV2 cell viability (46). BV2/shCtrl and BV2/shAnxa2 cells were seeded in 96-well plates at the density of 1 × 10^4^ cells/well overnight. The cells were subjected to OGD/R for 24 h. During the last 2 h of culture, 10 μl of CCK-8 solution was added into each well, and the absorbances were measured at 450 nm using a microplate reader.

3-(4,5-dimethylthiazolyl-2)-2,5-diphenyltetrazolium bromide (MTT) assay was performed to examine primary neuron viability according to the manufacturer’s protocol. Briefly, primary neurons (3 × 10^4^ cells/well) were seeded into 96-well plates and subjected to OGD and the supernatant of BV2 cells with OGD/R treatment. Ten microliters of MTT solution was added to each well and the plates were incubated at 37 °C for 4 h. Then the culture medium was completely removed, and 100 μl DMSO was added to each well to dissolve the formazan for 10 min in the shaker. The absorbances were measured at 490 nm using a microplate reader.

### Lactate Dehydrogenase Assay

The lactate dehydrogenase (LDH) release assay was used to assess cell damage (46). Briefly, BV2 microglial cells and neurons were seeded into the 24-well culture plates. Then, the supernatant of BV2 cells was collected after OGD/R. The level of LDH in the culture supernatant of cells was measured using the LDH-Cytotoxicity Assay Kit (Promega) according to the manufacturer’s protocol. The LDH release of the control group was considered as 100%.

### Immunofluorescence and TUNEL Staining

For the immunofluorescence assay (47), primary and BV2 microglial cells were grown on 12 mm glass coverslips in 24-well plates at a seeding density of 1 × 10^5^ cells/well. Cells were washed briefly in 1 × PBS and then fixed with 4% paraformaldehyde for 20 min at room temperature. Fixed cells were then permeabilized with 0.25% Triton X-100 in PBS for 10 min and treated with blocking buffer (1 × PBS, 3% BSA, and 0.1% Tween-20) for 1 h at room temperature. Cells were incubated with primary antibodies in blocking buffer overnight at 4 °C and washed with 1 × PBS supplemented with 0.1% Tween-20. They were subsequently incubated with fluorescence-conjugated secondary antibodies in blocking buffer for 2 h at room temperature and washed with 1 × PBS supplemented with 0.1% Tween-20 three times. Coverslips were mounted with Fluoromount-G mounting medium (Southern Biotechnology Associates, 0100-01). The images were captured using a fluorescence microscope.

To detect neuronal apoptosis (50), the TUNEL apoptosis assay was performed. Briefly, primary neuron cells were fixed in 4% formaldehyde for 20 min and then incubated with 0.1% Triton X-100 for 10 min at 37 °C. After washing 3 times with PBS, the cells were incubated with 50 μl TUNEL labeling solution for 3 h at 37 °C. Cells were washed 3 times with PBS. After that, cells were incubated overnight with anti-NeuN (1:500, Millipore, ABN78) antibody at 4 °C. They were washed 3 times with PBS and then incubated with the secondary antibodies conjugated to Alexa Fluor 555 (1:1000, Thermo Fisher Scientific) for 2 h at room temperature. After washing with PBS, they were incubated with 4′,6′-diphenyl-2-phenylindole for 5 min at room temperature and fixed with Fluoromount-G mounting medium. Stained neurons were chosen randomly, and images were taken using a confocal microscope.

### Quantification and Statistical Analysis

Images were acquired using a Leica SP8 confocal microscope. Images were analyzed using ImageJ software. For the p65 nuclear translocation assay, six fields of view per group were analyzed. The percentage of positive nuclear NF-κB p65 was calculated. For line scan profiling of fluorescence intensity analysis, images in each channel were separately thresholded, and the fluorescence distribution was measured in the overlapped region between two channels. The mean fluorescence intensities of Anxa2 for primary microglia and Anxa2 in Iba1-positive cells of brain tissues were measured. All values were shown as the mean ± SEM from at least three independent biological replicates. All data were analyzed using GraphPad Prism 8. Statistical significance was calculated by unpaired student’s *t* test for comparisons between two groups or two-way ANOVA for multiple comparisons. ∗*p* < 0.05, ∗∗*p* < 0.01, ∗∗∗*p* < 0.001, ∗∗∗∗*p* < 0.0001 were defined as statistically significant.

## Results

### Quantitative Proteome and Phosphoproteome Revealed the Immune and Inflammatory Response Involved in the Progression of Cerebral I/R

To characterize the cellular changes in cerebral I/R injury, adult mice were subjected to ischemia (MCAO) 1.5 h and reperfusion 24 h or sham surgery. TTC staining confirmed a significant increase in the infarct volume by more than 40% compared with the sham group 24 h post-I/R (supplemental Fig. S1, *A* and *B*). This procedure markedly reduced cerebral blood flow to 70 to 80% during MCAO, which subsequently recovered by almost 70% after reperfusion compared with the sham group (supplemental Fig. S1, *C* and *D*). Next, to assess cerebral I/R-induced neuronal death in the hippocampus, we performed immunohistochemical staining and observed an approximately 35% reduction in the number of NeuN-positive neurons in the CA1 region following I/R, compared to the sham group (supplemental Fig. S1, *E* and *F*). These results demonstrated that cerebral I/R also induced cerebral infarction and significant death of hippocampal neurons.

**Fig. 1:**
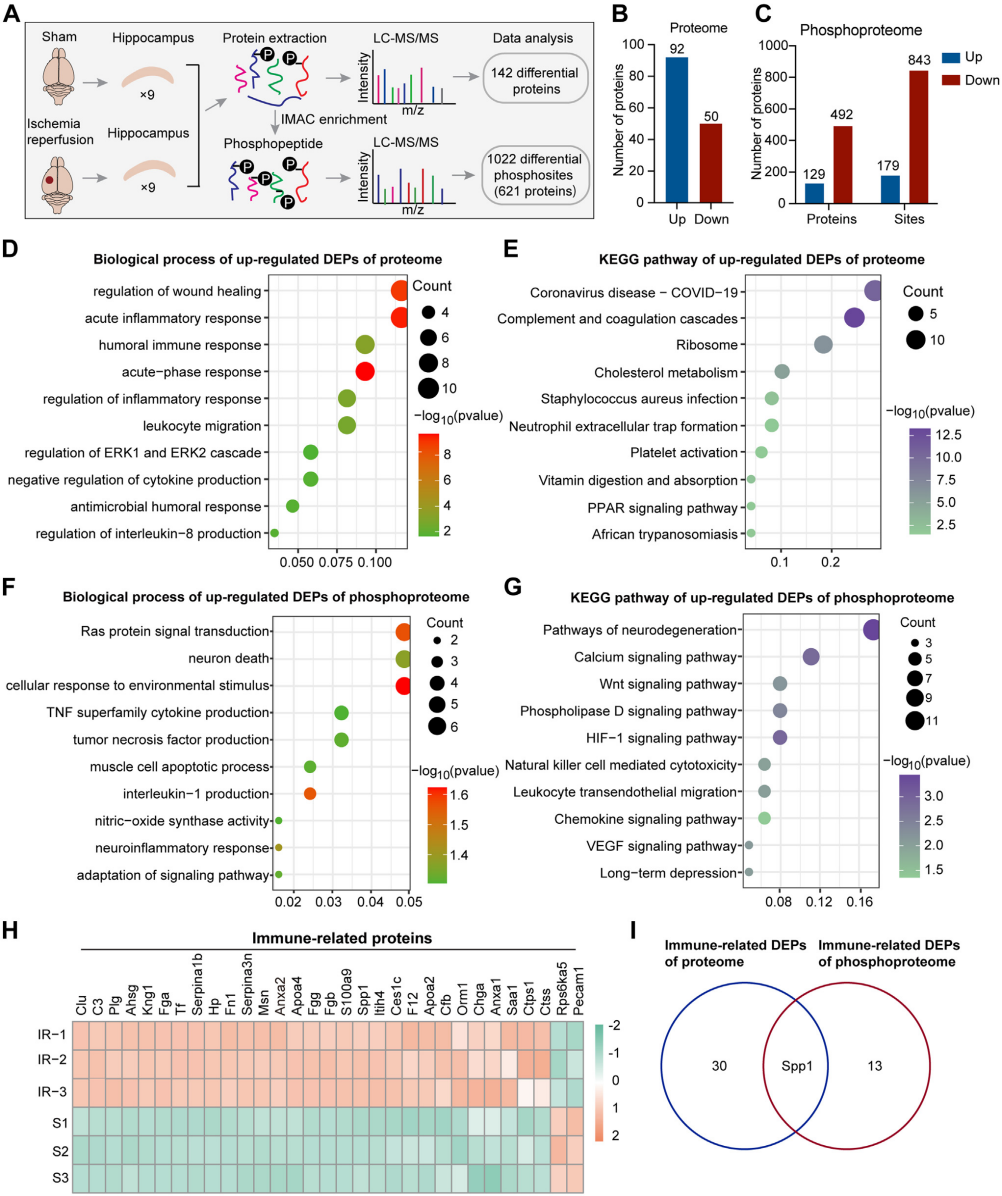
**Quantitative proteome and phosphoproteome revealed the immune inflammatory response in cerebral ischemia reperfusion.***A*, schematic diagram of proteome and phosphoproteome profiles. Three samples of hippocampi for each replicate after sham operation or ischemia 90 min reperfusion 24 h were analyzed by LC-MS/MS. *B* and *C*, the number of differentially expressed proteins (DEPs) in proteome (*B*) or phosphoproteome (*C*) between cerebral I/R *versus* sham groups (fold change >1.2 or <1/1.2, *p*-value <0.05). *Blue* represents upregulated proteins and *red* represents downregulated proteins. *D*–*G*, biological process analyses (*D* and *F*) and KEGG pathway analyses (*E* and *G*) of upregulated DEPs after cerebral I/R in proteome (*p*-value <0.05) (*D* and *E*) or phosphoproteome (*p*-value <0.05) (*F* and *G*). *H*, cluster analysis of the immune inflammatory-related proteins significantly altered after cerebral I/R in proteome. The column on the *left* and the row lower listed the replicate samples and the names of immune-related proteins, respectively. *Orange* and *green* colors represent upregulated and downregulated genes, respectively. *I*, Venn diagram of immune-related DEPs between proteome and phosphoproteome. DEP, differentially expressed protein; I/R, ischemic reperfusion; KEGG, Kyoto Encyclopedia of Genes and Genomes; MS/MS, tandem mass spectrometry.

To investigate the potential molecular mechanism of neuronal death in the progression of cerebral I/R injury, we used the TMT labeling technique, immobilized metal affinity chromatography enrichment, and high-resolution LC-MS/MS analysis to obtain the quantitative proteome and phosphoproteome from hippocampal tissues isolated from cerebral ischemia 1.5 h and reperfusion for 24 h and sham-operated mice (Fig. 1*A*). In total, 4426 proteins were quantified. We identified the 142 DEPs, among which 92 proteins were upregulated, and 50 proteins were downregulated (*p*-value <0.05) after cerebral I/R compared to the sham group (Fig. 1*B* and supplemental Table S1). In the phosphoproteome, a total of 4462 phosphosites from 1394 proteins were quantified. We identified 179 phosphorylation sites of 129 proteins that were upregulated and 843 phosphorylation sites of 492 proteins that were downregulated after cerebral I/R as compared with the sham group (Fig. 1*C* and supplemental Table S2). To further identify the biological role and functional annotation of DEPs obtained by the quantitative proteome and phosphoproteome, we performed GO enrichment analysis in the DEPs between I/R *versus* sham. The results showed that inflammatory-related biological processes were significantly enriched in the upregulated proteins, such as acute inflammatory response, humoral immune response, leukocyte migration, and regulation of cytokine production (Fig. 1*D* and supplemental Table S3). Consistently, KEGG pathway analyses for upregulated proteins showed that immune inflammatory-related pathways such as Coronavirus disease, complement and coagulation cascades, and *Staphylococcus aureus* infection were also enriched (Fig. 1*E* and supplemental Table S3). Similarly, GO and KEGG pathway analyses were performed for the differentially expressed phosphorylated proteins. Upregulated phosphorylated proteins were also enriched in some immune inflammatory-related biological processes and pathways (Fig. 1, *F* and *G*, and supplemental Table S4), indicating that immune inflammatory response is an important biological event altered during cerebral I/R. In contrast, for the downregulated phosphorylated proteins, we observed enrichment of synaptic transmission-related biological processes and pathways (supplemental Fig. S2, *A*–*D* and supplemental Table S4), suggesting significant synaptic abnormalities are associated with cerebral I/R before neuronal death. Collectively, these results indicate that the activation of the immune-inflammatory response triggered by cerebral I/R may lead to synaptic dysfunction and neuron injury.

Furthermore, the heatmap showed 31 immune-related DEPs, including 29 upregulated and 2 downregulated proteins after cerebral I/R (Fig. 1*H*). We also obtained 14 differentially phosphorylated proteins and 24 phosphorylated sites associated with immune inflammatory response (supplemental Figs. S2*E*, and S3). Interestingly, we found Spp1 (also known as Osteopontin [OPN]), a secreted multifunctional glycophosphoprotein that is involved in various biological processes, including inflammation, immune response, cell survival, cell adhesion, and neovascularization (51, 52, 53, 54, 55), was significantly enriched in the common immune-related DEPs of both proteome and phosphoproteome (Fig. 1*I*). Additionally, Spp1 expression is increased in ischemic stroke and exerts a neuroprotective effect (56, 57, 58). Consistently, previous studies have shown that the Spp1 function is highly regulated by post-translational modifications, such as protein phosphorylation (59). There are 36 phosphorylation sites in human Spp1 identified to date (55, 60, 61). Our results showed that Spp1 expression was significantly upregulated in the hippocampus after cerebral I/R (Fig. 1H). Moreover, Spp1 phosphorylation at Thr166, Ser231, and Ser250 were significantly upregulated in the hippocampus following cerebral I/R (Fig. 1*I*, supplemental Fig. S2*E*, and supplemental Table S5).

### Combinatorial Analyses of Transcriptomic, Proteomic, and Phosphoproteomic Profiles Revealed Anxa2 as a Key Molecule in Cerebral I/R-Triggered Inflammatory Response

**Fig. 2:**
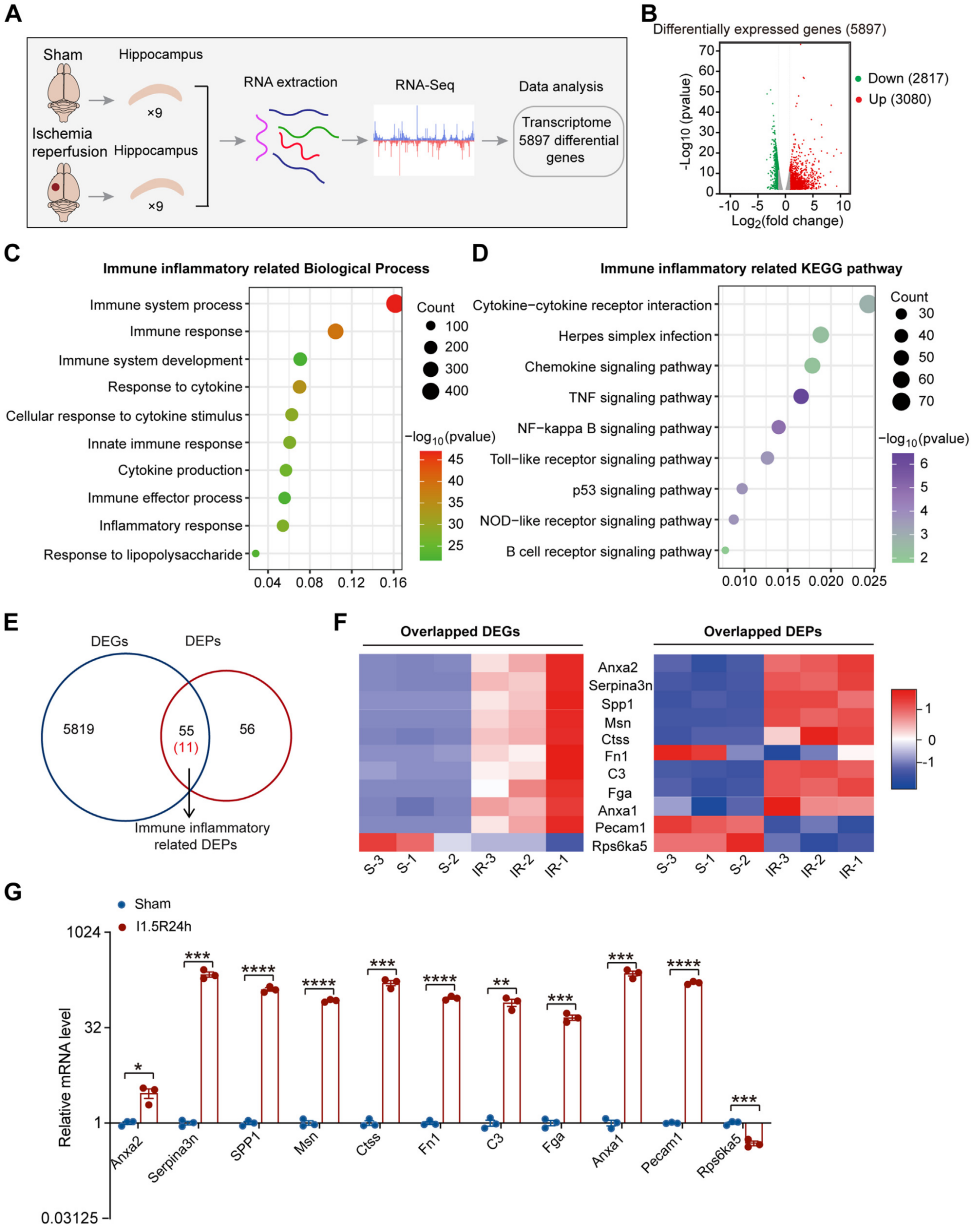
**Combinatorial analyses of transcriptome, proteome, and phosphoproteome profiles revealed Anxa2 was related to I/R-triggered inflammatory response.***A*, schematic diagram of transcriptome profile. Three samples of hippocampi for each replicate after sham operation or ischemia 90 min reperfusion 24 h were analyzed by RNA-seq. *B*, volcano plot of the differential gene expression between sham and I/R groups (fold change >1.2 or <1/1.2, *p*-value <0.05). *C* and *D*, top-ranked biological processes (*C*) and KEGG pathways (*D*) related to immune inflammatory responses enriched for differentially expressed genes (DEGs) after I/R in transcriptome profile (*p*-value <0.05). *E*, Venn diagram of common DEGs and DEPs associated with the immune inflammatory response between transcriptome and proteome. *F*, heatmap of the expression trends of common DEGs and DEPs related to immune inflammatory responses between I/R *versus* sham. Normalized and z-scored (color-coded) value of log2 PFKM of each gene or log2 expression value of each protein is shown. *Red* and *blue* colors represent upregulated and downregulated genes, respectively. *G*, the expression level of immune inflammatory-related genes was analyzed by RT-PCR after I/R relative to Sham. GAPDH was used as the internal control for RT-PCR. Data are presented as means ± SEM, Statistical significance was determined by unpaired student’s t-tests, ∗*p* < 0.05, ∗∗*p* < 0.01, ∗∗∗*p* < 0.001, ∗∗∗∗*p* < 0.0001. Anxa2, annexin A2; DEP, differentially expressed protein; I/R, ischemic reperfusion; KEGG, Kyoto Encyclopedia of Genes and Genomes.

To identify gene expression changes in response to cerebral I/R injury, we performed RNA-seq and transcriptomic profiling of hippocampal tissues isolated from sham-operated mice or those subjected to 1.5 h of ischemia, followed by 24 h of reperfusion (Fig. 2*A*). PCA of the RNA-seq data showed two distinct clusters, indicating that the gene expression pattern between these two groups was different from each other (supplemental Fig. S4*A*). A total of 5897 DEGs (3080 upregulated and 2817 downregulated DEGs) were obtained in I/R *versus* sham (Fig. 2*B*, and supplemental Table S6). GO analysis revealed that many of the upregulated genes were enriched in the immune inflammation processes after cerebral I/R (Fig. 2*C* and supplemental Table S7). Similarly, the KEGG pathway analysis for those upregulated genes also showed enrichment in immune inflammatory response–related pathways, including TNF-α signaling, NF-κB signaling, cytokine-cytokine receptor interaction, NOD-like receptor signaling, and Toll-like receptor signaling (Fig. 2*D*, and supplemental Table S7). These data demonstrated that immune inflammatory responses play a significant role in cerebral I/R injury.

We next focused on the significantly altered immune-related genes in cerebral I/R injury for subsequent analysis. First, we intersected DEGs and DEPs and found 66 commonly regulated molecules (supplemental Table S8). Eleven of them belong to the immune inflammatory responses–related genes/proteins (Fig. 2, *E* and *F*, and supplemental Fig. S5). Protein-protein interaction network analysis for the 11 common genes/proteins identified Anxa2 as an integral component of the protein hub (supplemental Fig. S4*B*). Next, we performed RT-qPCR to validate the expression levels of the 11 common immune inflammatory-related genes in hippocampal tissues of cerebral I/R and sham-operated mice and found results consistent with the RNA-seq data (Fig. 2*G*).

### Upregulation and Activation of Anxa2 in Microglia Cells upon OGD/R

**Fig. 3:**
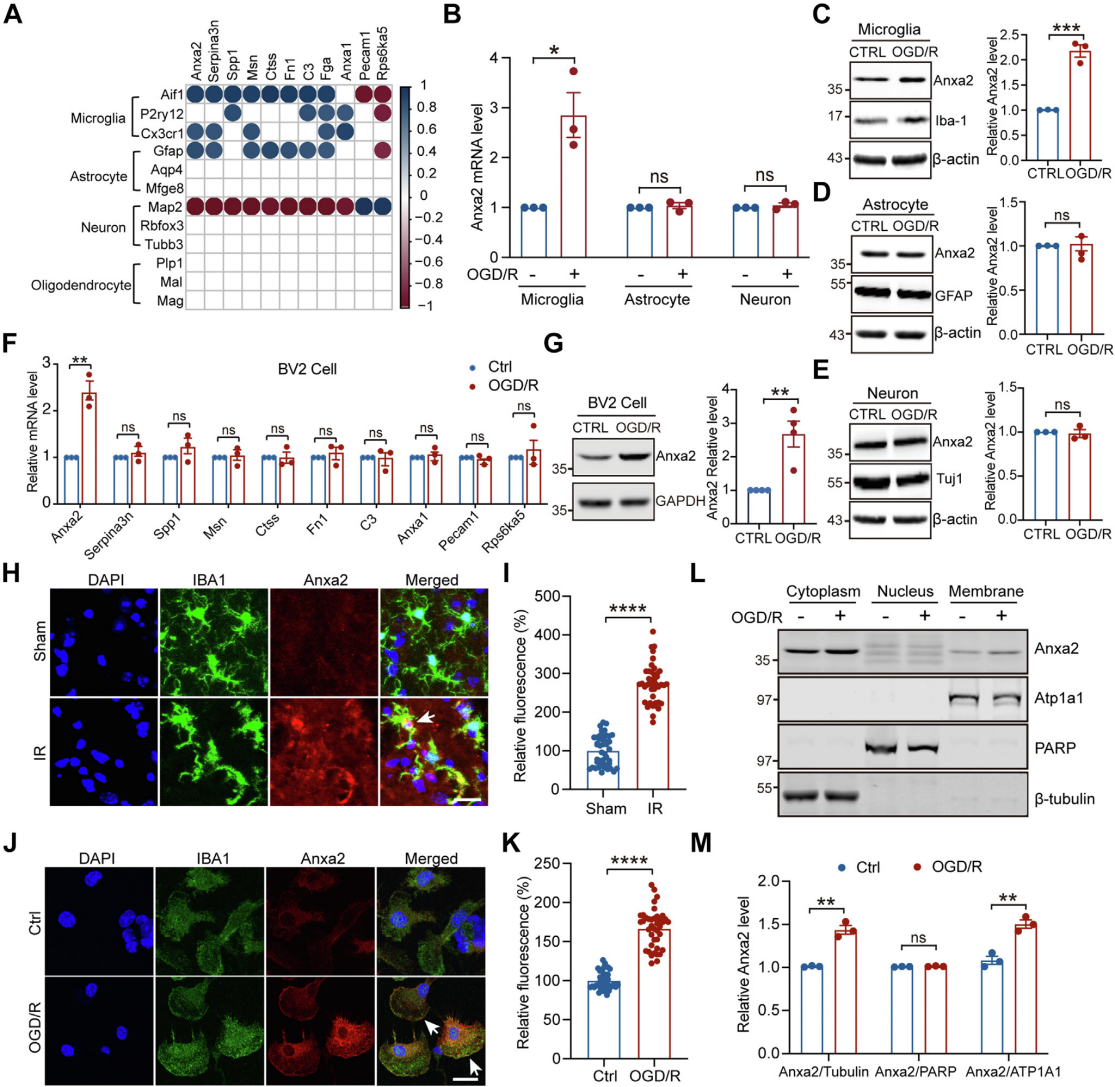
**Anxa2 was specifically upregulated and translocated onto the membrane in microglia cells upon OGD/R.***A*, Pearson's correlation analysis between 11 common DEGs/DEPs identified by transcriptome and proteome and the marker genes of different brain cell types including microglia, astrocytes, neurons, and oligodendrocytes. The column on the *left* listed the marker gene names of microglia, astrocytes, neurons, and oligodendrocytes, respectively. The row on the top listed the gene names of 11 common DEGs/DEPs. *Blue* and *red* colors represent positive and negative, respectively. *p*-value <0.05. *B*, Anxa2 mRNA level detected by RT-PCR in primary microglia, astrocytes, and neurons after OGD/R. *C*–*E*, representative blot images and quantitative analysis of Anxa2 protein level after OGD/R in primary microglia (*C*), astrocyte (*D*), and neuron (*E*) detected by Western blot. *F*, mRNA level of the immune inflammatory responses-genes (11 common DEGs/DEPs) after OGD/R in BV2 microglia cells. *G*, representative blot images and quantitative analysis of Anxa2 expression level after OGD/R in BV2 microglia cells detected by Western blot. *H*, representative double immunostaining of Anxa2 (*red*) with Iba-1 (a microglial marker, *green*) in brain tissue after I/R surgery. *White arrows* showing Anxa2 colocalization with Iba-1. *I*, quantification of Anxa2 fluorescence intensity in Iba1-positive cells was quantified using ImageJ. The analytical graph was presented as normalized to the sham group. Scale bars represent 20 μm. The mean ± SEM is displayed for all data. ∗∗∗∗*p* < 0.0001. *J*, representative double immunostaining of Anxa2 (*red*) with Iba-1 (*green*) in primary microglial cells after OGD/R. *White* arrows showing Anxa2 translocation to the membrane. *K*, quantification of Anxa2 fluorescence intensity was quantified using ImageJ. The analytical graph was presented as normalized to the control group. Scale bars represent 20 μm. ∗∗∗∗*p* < 0.0001. *L* and *M*, BV2 cells were treated with OGD/R and then performed subcellular fraction assay to isolate the cytoplasmic, nuclear, and membrane fractions. Representative blot images (*L*) and quantitative analysis of Anxa2 protein expression levels detected by Western blot. The quantitative analysis (*M*) shows that Anxa2/β-tubulin in the cytoplasm, Anxa2/PARP in the nucleus, and Anxa2/ATP1A1 at the membrane. Data are presented as means ± SEM, Statistical significance was determined by unpaired student’s t-tests, ∗*p* < 0.05, ∗∗*p* < 0.01, ∗∗∗*p* < 0.001, ∗∗∗∗*p* < 0.0001. Anxa2, annexin A2; DEG, differentially expressed gene; DEP, differentially expressed protein; I/R, ischemic reperfusion; OGD/R, oxygen-glucose deprivation and reoxygenation.

To further characterize the role of Anxa2 in cerebral I/R injury, we performed Pearson's correlation analysis of the 11 immune response–related proteins with the markers of different brain cell types and observed high correlations of Anxa2 protein expression with microglia, astrocytes, and neurons, but with oligodendrocytes (Fig. 3*A*). We then determined the gene and protein expressions of Anxa2 in these cell types following OGD/R, a well-established *in vitro* model of cerebral I/R (62) and found that OGD/R significantly upregulated Anxa2 mRNA (Fig. 3*B*) and protein levels only in primary microglial cells (Fig. 3, *C*–*E*).

Previous studies demonstrated that microglia are immune cells that reside in the brain and play an important role in regulating brain inflammation after cerebral I/R (22, 63, 64). To identify the function of those 11 immune response genes in microglia during OGD/R, we used BV2-immortalized mouse microglial cells (65) subjected to OGD/R treatment and found that only *Anxa2* mRNA expression was robustly upregulated compared to that of control BV2 cells (Fig. 3*F*). Consistently, immunoblotting assays also showed that only Anxa2 protein expression (Fig. 3*G*), but not the other common immune-inflammatory response–associated proteins, was significantly upregulated in OGD/R-treated BV2 microglia cells (supplemental Fig. S6). Importantly, immunohistochemical staining of hippocampal tissues of the mouse brain following cerebral I/R injury also showed a robust upregulation of Anxa2 protein expression in Iba1-positive microglial cell population (Fig. 3, H and I). These results demonstrated that the expression of Anxa2 is specifically regulated in microglial cells in response to OGD/R *in vitro* and cerebral I/R *in vivo*.

Anxa2 contains an N-terminal hydrophilic tail and four Ca^2+^-binding annexin repeats. It forms a heterotetrameric complex with S100A10 protein, which enhances the binding affinity of Anxa2 to Ca^2+^ and promotes Anxa2 translocation to the plasma membrane (66). To examine whether OGD/R treatment affects Anxa2 translocation, we performed immunostaining of BV2 cells in response to OGD/R and found a significant increase in the overall Anxa2 protein expression compared to control BV2 cells (Fig. 3, *J* and *K*). Interestingly, Anxa2 immunoreactivity could be readily detected on the plasma membrane, indicating the activation and translocation of Anxa2 to the plasma membrane following OGD/R (Fig. 3*J*). In consistent with our immunostaining results, the subcellular fraction assay also showed significant increases in the levels of Anxa2 in the cytoplasm and the cell membrane (Fig. 3, *L* and *M*). These findings demonstrated that Anxa2 is activated and accumulated on the membrane of microglial cells after OGD/R.

### Anxa2 Knockdown Impairs the Expression of Inflammatory Response Genes in BV2 Microglial Cells upon OGD/R

**Fig. 4:**
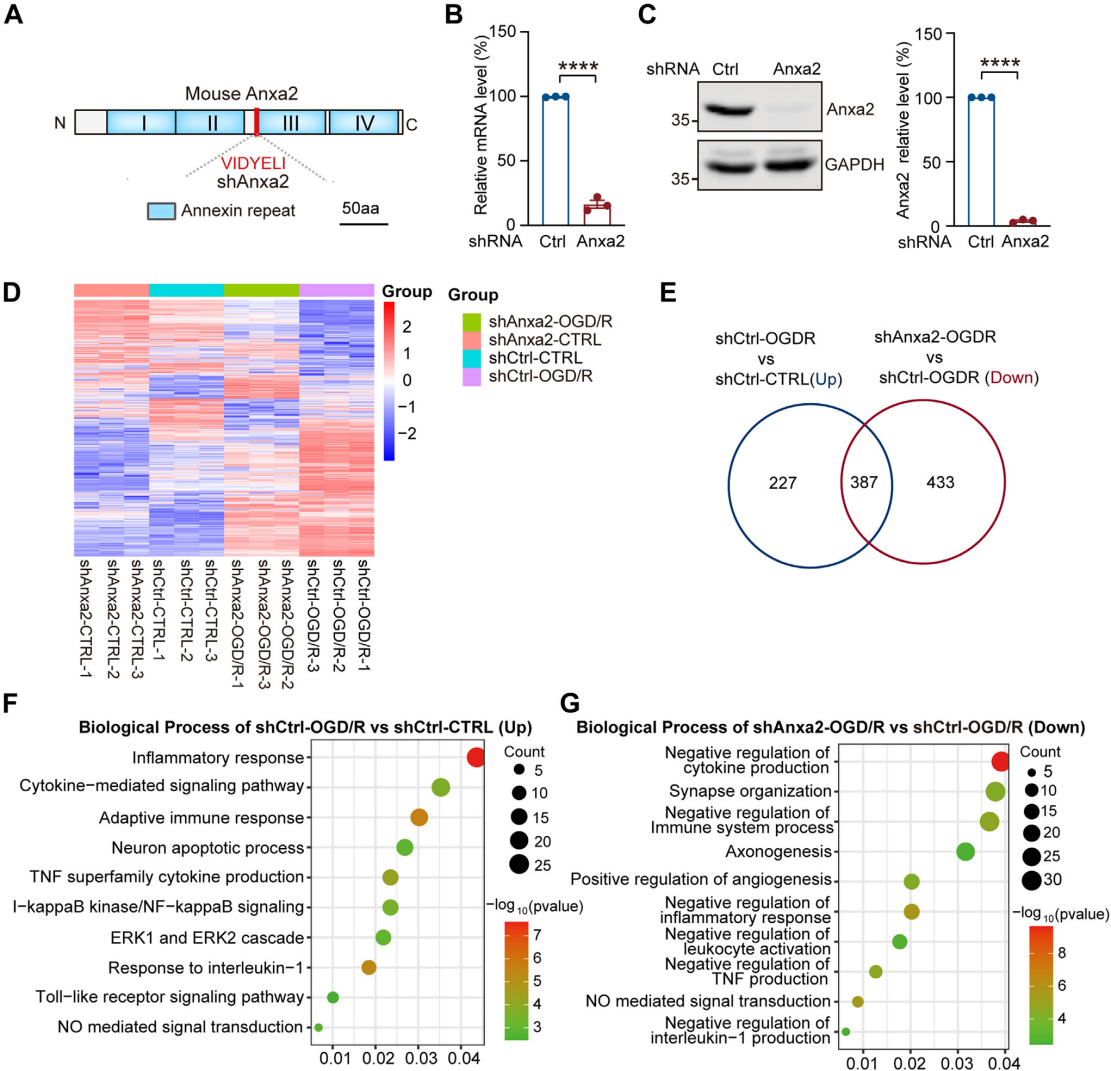
**Knockdown of Anxa2 affected the inflammatory response genes and pathways upon OGD/R in BV2 microglial cells.***A*, schematic of Anxa2 encoding region and target site of a stable cell line of Anxa2 knockdown by small hairpin RNA (shRNA) in BV2 cells. *B*, RT-PCR analysis for Anxa2 mRNA expression in BV2 stable cell lines of small hairpin RNA control (shCtrl) and small hairpin RNA of Anxa2 gene (shAnxa2), respectively. *C*, representative blot images and quantitative analysis of exogenous Anxa2 protein in shCtrl and shAnxa2 BV2 stable cell lines detected by Western blot. Data are presented as means ± SEM. Statistical significance was determined by unpaired Student’s t-tests. ∗∗∗∗*p* < 0.0001. *D*, differential gene expression volcano plots and (*E*) Venn diagram revealed significant changes in BV2 microglial cells transcriptome of Anxa2 knockdown after OGD/R (*p*-value <0.05, fold change >1.5). *F*, biological process analyses of DEGs upregulated in shCtrl-group after OGD/R (*p*-value <0.05). *G*, the biological process of DEGs downregulated in shAnxa2-OGD/R *versus* shCtrl-OGD/R group (*p*-value <0.05). Anxa2, annexin A2; DEG, differentially expressed gene; OGD/R, oxygen-glucose deprivation and reoxygenation.

To investigate the roles of Anxa2 in microglial cells after OGD/R, we generated a stable BV2 cell line that expresses a specific shRNA that targets the *Anxa2* transcript (Fig. 4*A*). RT-qPCR and Western blot analyses confirmed robust suppression of Anxa2 mRNA and protein expression with more than 90% efficiency (Fig. 4, *B* and *C*). We next performed transcriptomic profiling of BV2 stable cell lines expressing control shRNA (shCtrl) or Anxa2 shRNA (shAnxa2) without or with OGD/R treatment. The heatmap (Fig. 4*D*) and PCA analyses (supplemental Fig. S7*A*) of the RNA-seq data showed that the gene expression pattern among these four groups was distinct. In subsequent analyses, we mainly compared OGD/R-induced DEGs in control and shAnxa2 BV2 cells. Our data revealed that 387 genes that were normally upregulated in control cells upon OGD/R treatment were significantly downregulated in shAnxa2 BV2 cells (Fig. 4*E*, and supplemental Table S9). Interestingly, GO and KEGG enrichment analyses revealed that OGD/R-activated immune-inflammatory response–related biological pathways were also downregulated in Anxa2 knockdown cells (Fig. 4, *F* and *G*, supplemental Fig. S7, *B* and *C*, and supplemental Table S10). We then performed heatmap plot and STRING protein-protein interaction network analysis to obtain the inner interactions for these immune-related DEGs after Anxa2 knockdown with OGD/R treatment (supplemental Fig. S7, *D* and *E*). These data indicated that Anxa2 affects the expression of inflammation-related genes in microglia, further supporting its involvement in OGD/R-induced injury.

### Anxa2 Knockdown Ameliorates Immune Inflammatory Response and Apoptosis Induced by OGD/R in BV2 Microglial Cells

**Fig. 5:**
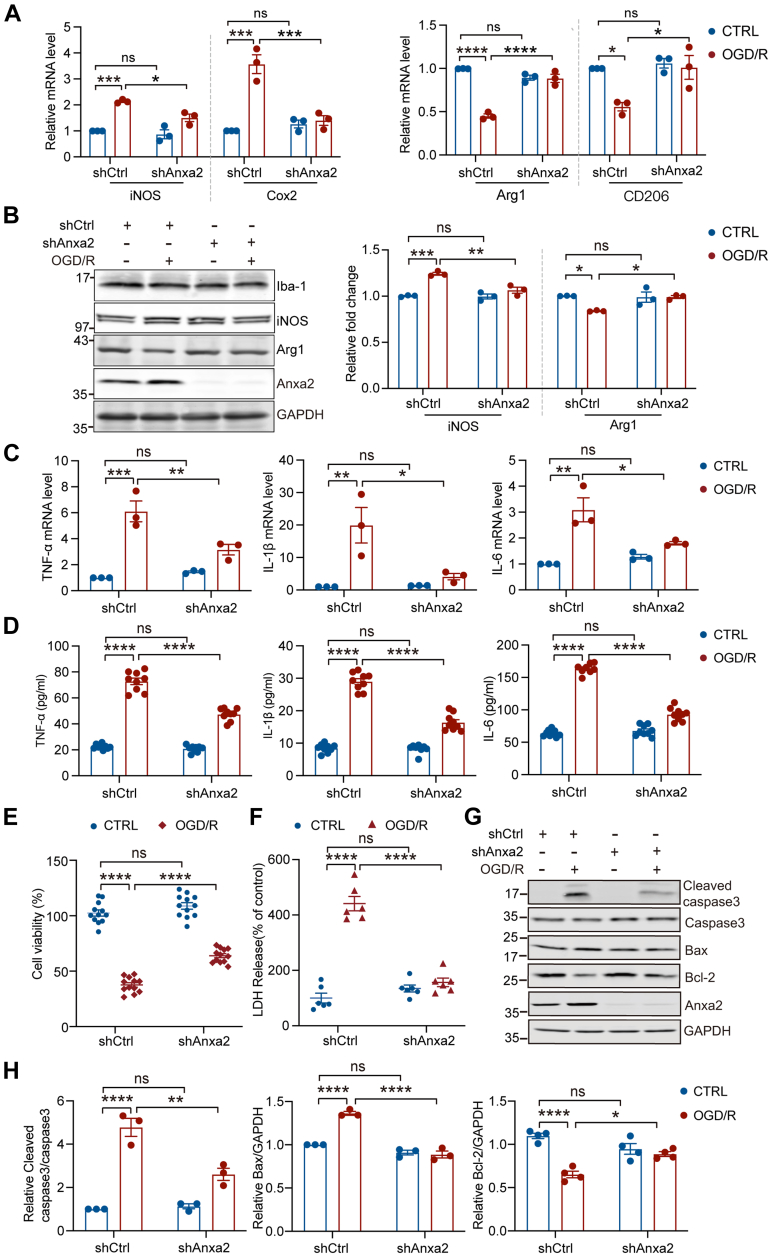
**Anxa2 knockdown ameliorated immune inflammatory response and apoptosis of BV2 microglial cells induced by OGD/R.***A*, the mRNA expression levels of pro-inflammatory marker genes iNOS and COX2 and anti-inflammatory marker genes Arg1 and CD206 in shCtrl and shAnxa2 BV2 cells with or without OGD/R treatment. *B*, representative blot images and quantitative analysis of iNOS and Arg1 protein expression levels detected by Western blot in shCtrl and shAnxa2 BV2 cells with or without OGD/R treatment. GAPDH was an internal normalized control. *C*, the relative mRNA levels of inflammatory mediators TNF-α, IL-1β, and IL-6 in shCtrl or shAnxa2 BV2 cells after OGD/R by RT-PCR. *D*, the expression levels of TNF-α, IL-1β, and IL-6 in supernatants of shCtrl or shAnxa2 BV2 cells after OGD/R were measured by ELISA assay. *E*, cell viability accessed by CCK8 assay in shCtrl or shAnxa2 BV2 cells after OGD/R. *F*, cell death accessed by LDH release in shCtrl or shAnxa2 BV2 cells after OGD/R. *G* and *H*, representative blot images and quantitative analysis of apoptosis-related proteins cleaved caspase3, Bax, and Bcl2 in shCtrl or shAnxa2 BV2 cells after OGD/R detected by Western blot. All data were shown as mean ± SEM. Statistical significance was determined by two-way ANOVA for multiple comparisons. ∗*p* < 0.05, ∗∗*p* < 0.01, ∗∗∗*p* < 0.001. ∗∗∗∗*p* < 0.0001. All experiments were repeated at least three times. Anxa2, annexin A2; IL-6, interleukin-6; IL-1β, interleukin-1beta; iNOS, inducible nitric oxide synthase; LDH, lactate dehydrogenase; OGD/R, oxygen-glucose deprivation and reoxygenation; TNF-α, tumor necrosis factor-α.

Microglia exhibit pro- and anti-inflammatory responses according to environmental stimuli (67). To determine the role of Anxa2 in regulating immune inflammatory response, we performed RT-qPCR in BV2 cells following OGD/R and found that Anxa2 knockdown attenuated the expression of pro-inflammatory mediators prostaglandin-endoperoxide synthase 2 (COX2) and inducible iNOS and inhibited the OGD/R-induced downregulation of anti-inflammatory mediators arginase 1 (Arg1) and macrophage mannose receptor 1 (CD206) (Fig. 5*A*). These findings were further corroborated at the protein levels with Western blotting analyses (Fig. 5*B*). Given that iNOS and Arg-1 are two important enzymes in the arginine metabolism pathway, these results reflect the polarization of microglia phenotypes (68). Our findings indicated that Anxa2 knockdown dysregulates the expression pattern of pro- and anti-inflammatory factors in microglia in response to OGD/R. To further investigate the impact of Anxa2 knockdown on inflammatory response after OGD/R, we examined the expression of pro-inflammatory cytokines in BV2 cells. Upon OGD/R, the pro-inflammatory cytokines TNF-α, IL-1β, and IL-6 were robustly upregulated at both mRNA (Fig. 5*C*) and protein (Fig. 5*D*) levels in BV2 microglia cells. In contrast, the regulation of their expression was significantly attenuated in Anxa2 knockdown BV2 cells. Collectively, these results indicated that Anxa2 acts as an upstream activator of the inflammatory response during OGD/R.

We next investigated whether Anxa2 knockdown can affect the survival of BV2 microglia cells after OGD/R. By using a colorimetric cell survival assay, we found that the OGD/R treatment significantly reduced cell viability. Notably, Anxa2 knockdown increased the cell viability after OGD/R compared to the control cells (Fig. 5*E*). Consistently, we also observed that the OGD/R treatment drastically increased LDH release that reflects decreased cell viability (44), while Anxa2 knockdown suppressed the increase in LDH release (Fig. 5*F*). The activation of caspase-3 and B-cell lymphoma 2–associated X protein (Bax) and the decreased production of B-cell lymphoma 2 (Bcl-2) are major biochemical markers of apoptotic cells (69). By examining the expression of these apoptotic markers after OGD/R in BV2 microglia cells, we found a significant decrease of the proapoptotic proteins caspase-3 and Bax, concomitant with an increase in the expression of anti-apoptotic protein Bcl-2 in shAnxa2 BV2 microglia cells after OGD/R (Fig. 5, *G* and *H*). Taken together, these results suggested that Anxa2 knockdown reduced microglial activation in response to OGD/R and ameliorated OGD/R-induced inflammatory response and cell apoptosis in BV2 microglial cells.

### Anxa2 Knockdown Inhibits NF-κB Signaling Activation Triggered by OGD/R in BV2 Microglial Cells

**Fig. 6:**
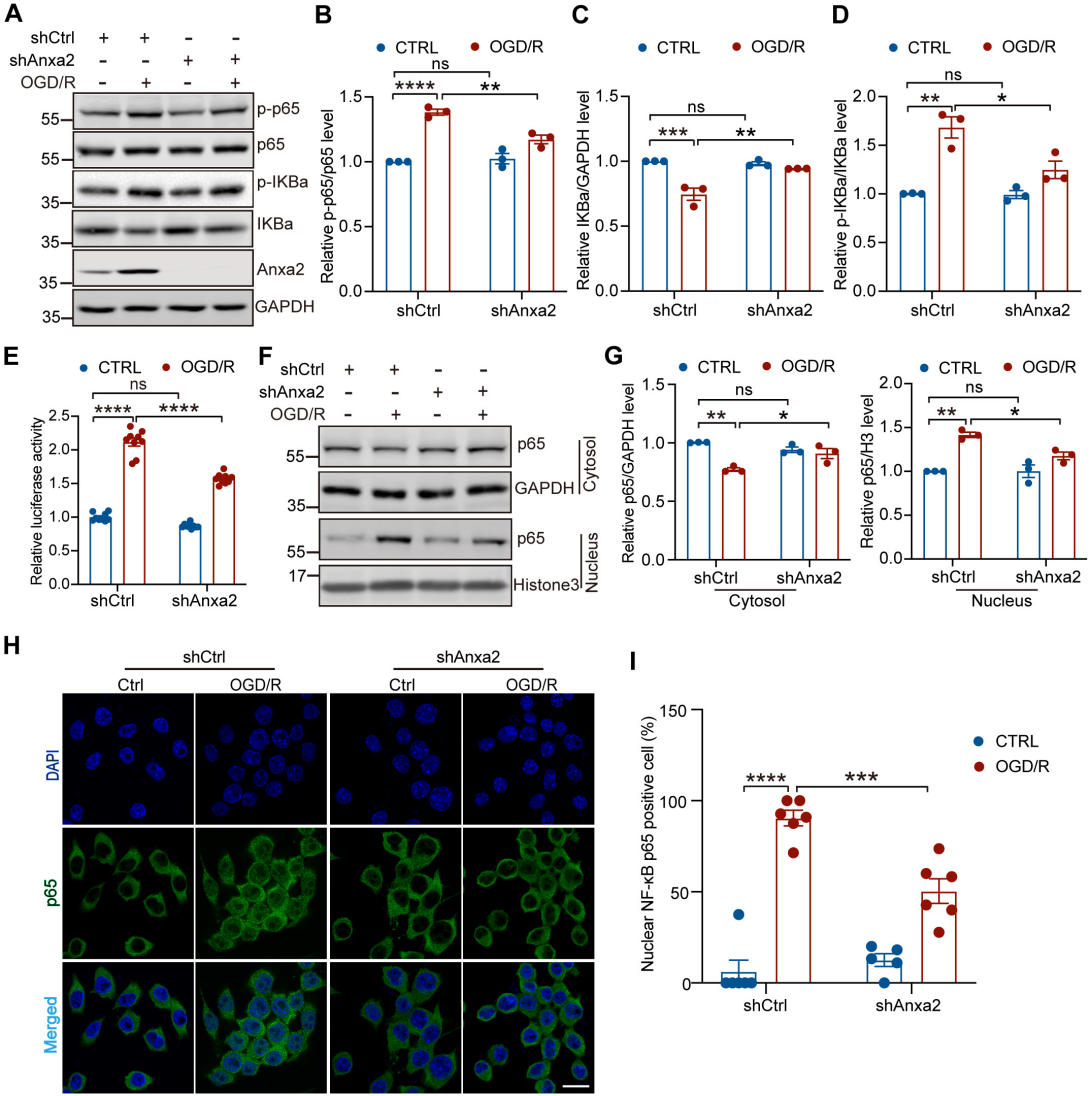
**Anxa2 knockdown inhibited NF-κB signaling activation in OGD/R-treated BV2 microglial cells.***A*–*D*, representative blot images and quantitative analysis of NF-κB signaling components including phosphorylated p65 (p-p65), total p65, phosphorylated IKBa, and total IKBa in shCtrl and shAnxa2 BV2 cells after OGD/R detected by Western blot. GAPDH was an internal normalized control. *E*, relative luciferase activity of NF-κB after Anxa2 knockdown in OGD/R-induced BV2 cells. *F* and *G*, representative blot images and quantitative analysis of the subcellular accumulated NF-κB p65 in the cytoplasmic and nuclear fractions, respectively, in shCtrl and shAnxa2 BV2 cells after OGD/R detected by Western blot. As a protein loading control, the data were normalized to GAPDH (cytoplasmic marker) or Histone H3 (nuclear marker). *H* and *I*, representative images (*H*) and quantitative analysis (*I*) of the positive nucleus localization of NF-kB p65 subunit accessed by immunofluorescence staining in shCtrl and shAnxa2 BV2 cells after OGD/R. Scale bar represents 20 μm. Data are shown as mean ± SEM. Statistical significance was determined by two-way ANOVA for multiple comparisons, ∗*p* < 0.05, ∗∗*p* < 0.01, ∗∗∗*p* < 0.001, ∗∗∗∗*p* < 0.0001. All experiments were repeated at least three times. Anxa2, annexin A2; NF-κB, nuclear factor-kappa B; OGD/R, oxygen-glucose deprivation and reoxygenation.

To determine the effect and mechanism of Anxa2 activation on microglial inflammation in response to OGD/R, we first explored a potential transcriptional program through which Anxa2 regulates the expression of TNF-α, IL-1β, and IL-6 in BV2 cells. Previous studies revealed that the NF-κB signaling pathway is activated, which causes an increase in the production of inflammatory mediators following ischemia (70). A recent study also shows that Anxa2 can directly regulate NF-κB activation by binding to the p50 subunit in pancreatic cancer cells (71). Moreover, Anxa2 knockdown reduces the transcriptional activity of NF-κB and downregulates nuclear translocation of p50 in the neuroblastoma cell line (72). Thus, we hypothesize that Anxa2 regulates the expression of pro-inflammatory cytokines by activating the NF-κB signaling in microglia. To test this hypothesis, we examined the levels of total and phosphorylated p65 subunit and IKβα by immunoblotting. We found that the phosphorylation level of the p65 subunit of NF-κB and IKβα were significantly increased upon OGD/R treatment in control BV2 cells, but they were inhibited in Anxa2 knockdown cells (Fig. 6, *A*, *B*, and *D*). Additionally, the protein level of IKβα was significantly downregulated after OGD/R, indicating that IKβα was degraded after OGD/R. However, OGD/R-induced downregulation of IKβα was prevented by the loss of Anxa2 expression (Fig. 6, *A* and *C*). In summary, our findings suggested that Anxa2 not only regulates the expression of NF-κB signaling but is also required to activate NF-κB signaling in microglia after OGD/R.

To examine whether OGD/R could affect the interaction between Anxa2 and the p50 subunit of NF-κB, we performed the co-immunoprecipitation assay and found that these two proteins formed a complex and that their interaction was not affected by OGD/R (supplemental Fig. S8). To further consolidate the relationship between Anxa2 and NF-κB, we determined the effects of Anxa2 on the transcriptional activity of NF-κB using a luciferase reporter assay. We found that OGD/R treatment enhanced the transcriptional activity of NF-κB, while Anxa2 knockdown significantly attenuated this process (Fig. 6*E*). Because activated NF-κB translocates into the nucleus to regulate the expression of the target genes (73), we sought to determine if Anxa2 knockdown affects NF-κB translocation following OGD/R. To do this, we separated the cytosol and nuclear fractions by differential centrifugation followed by Western blotting analysis. We observed that the level of p65 in the nuclear extract was elevated in control but not in Anxa2-deficient BV2 cells after OGD/R (Fig. 6, *F* and *G*). Similarly, we also observed a robust translocation of p65 into the nucleus of control cells following OGD/R treatment by immunostaining, but it was significantly attenuated in Anxa2 knockdown cells (Fig. 6, H and I). Taken together, these results suggest that Anxa2 partially regulates OGD/R-induced activation of immune-inflammatory response in microglia through promoting p65 nuclear translocation and the activation of the NF-κB signaling pathway.

### Microglial Anxa2 Knockdown Protects Neurons Against OGD/R-Induced Injury

**Fig. 7:**
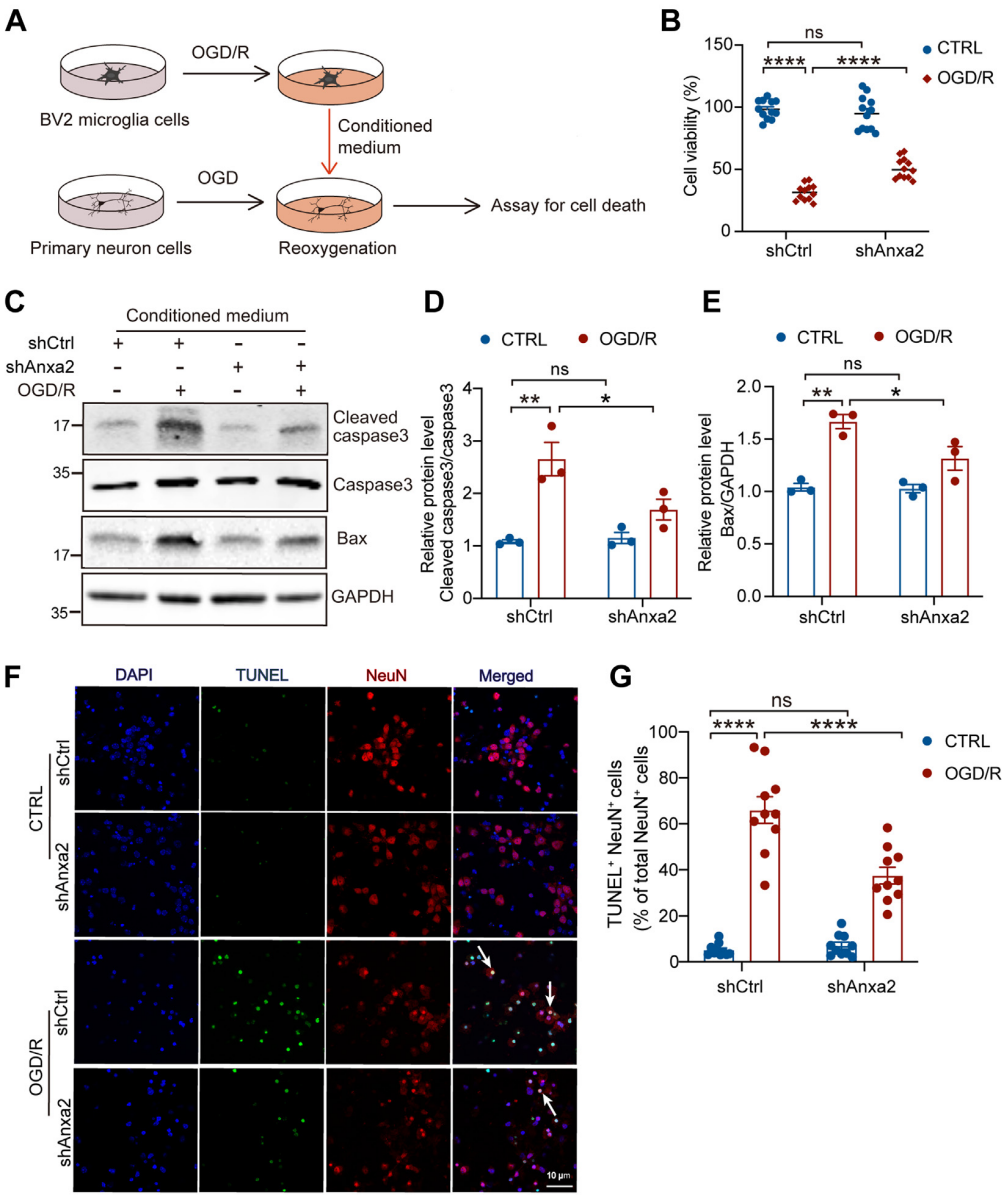
**Anxa2 knockdown alleviates OGD/R-induced neuronal injury.***A*, schematic diagram of primary neurons treated by BV2 microglia cells-conditional medium (CM) *in vitro* for the following assays. *B*, cell viability of primary neurons treated with conditional medium from shCtrl or shAnxa2 BV2 cells with or without OGD/R treatment using MTT assay. *C*–*E*, representative blot images (*C*) and quantitative analyses of cleaved caspase3 (*D*) and Bax (*E*) in neurons treated with conditional medium derived from shCtrl or shAnxa2 BV2 cells with or without OGD/R treatment. GAPDH was an internal normalized control. *F* and *G*, representative images (*F*) and quantitative analyses (*G*) of TUNEL (*green*) and NeuN (*red*) positive neurons subjected to OGD with conditional medium derived from shCtrl or shAnxa2 BV2 cells with or without OGD/R treatment. The arrow indicates the colocalization of TUNEL and NeuN. Scale bar represents 10 μm. n = 10. All data are shown as mean ± SEM. Statistical significance was determined by two-way ANOVA for multiple comparisons, ∗*p* < 0.05, ∗∗*p* < 0.01, ∗∗∗*p* < 0.001, ∗∗∗∗*p* < 0.0001. All experiments were repeated at least three times. Anxa2, annexin A2; MTT, 3-(4,5-dimethylthiazolyl-2)-2,5-diphenyltetrazolium bromide; OGD/R, oxygen-glucose deprivation and reoxygenation.

To investigate the neuroprotective effect of microglial Anxa2, we performed OGD on primary neurons for 90 min, after which they were treated with re-oxygenated conditioned medium derived from OGD/R-treated BV2 cells for 24 h *in vitro* (Fig. 7*A*). MTT assay revealed that the conditioned medium derived from Anxa2 knockdown-BV2 cells significantly decreased neuronal death (Fig. 7*B*). The incubation with re-oxygenated conditioned medium from Anxa2 knockdown BV2 cells markedly decreased the levels of cleaved Caspase-3 (Fig. 7, *C* and *D*) and the pro-apoptotic factor Bax (Fig. 7, *C* and *E*) compared to control conditions. Moreover, the TUNEL and NeuN double immunostaining assay showed that the re-oxygenated conditioned medium from Anxa2 knockdown BV2 cells significantly decreased the percentage of TUNEL-positive neurons compared to control neurons (Fig. 7, *F* and *G*). In summary, these data suggested that the loss of Anxa2 function in microglial cells nonautonomously protects neurons against OGD/R-induced neuron injury.

## Discussion

Ischemic stroke is caused by an inadequate supply of blood and oxygen due to the blockage of an artery. After vascular recanalization, restoring oxygen and blood flow can lead to oxidative stress, triggering inflammation that causes irreversible cell damage and death (74). Due to its clinical importance, various animal models have been developed to mimic human clinical stroke. In this study, we used a well-established MCAO and reperfusion model in mice. The gene expression of most molecules is closely related to the abundance of protein, and the function and biological roles of proteins are also regulated by posttranslational modifications (75). Proteomics is the comprehensive study of all proteins in intricate biological systems, and it has been applied to research on cerebral ischemia to identify blood biomarkers for diagnosis, therapeutic targets, and pathological mechanism studies. Nonetheless, most studies performed a single transcriptomic or proteomic profiling in isolation to reveal the dysregulation of the genes or proteins involved in biological processes and pathways. Those studies only examined some dysregulation genes or proteins by RT-PCR or Western blot while not exploring the cellular function and mechanism (76, 77, 78, 79).

The cortex, hippocampus, and striatum are extremely sensitive to cerebral ischemia in the brain. Neuronal cell death is induced by ischemic reperfusion in the mouse cortex, striatum, and hippocampus (80). Specifically, in human patients and experimental animal models with cerebral ischemia or hypoxia, CA1 pyramidal neurons are among the most vulnerable (81). We also found partial neuronal death in the CA1 area of the hippocampus. However, previous studies focus on the cortex in cerebral ischemia and reperfusion (76, 79). Therefore, we performed multi-omic profiling in the hippocampal tissue after cerebral I/R to identify key molecules and cellular mechanisms mediating neuronal death in the pathogenesis of cerebral I/R injury. Based on the integrated phosphoproteomic and proteomic profiling, we found that upregulated proteins and phosphorylated proteins were enriched in biological processes, such as acute inflammatory response, humoral immune response, regulation of ERK1 and ERK2 cascade, leukocyte migration, tumor necrosis factor production, interleukin-1 production, nitric-oxide synthase activity, and neuronal death. Furthermore, upregulated and phosphorylated proteins were enriched in the KEGG pathway, such as complement and coagulation cascades, calcium signaling pathways, natural killer cell–mediated cytotoxicity, and PPAR signaling pathways. In addition, we also enriched other biological pathways, including the regulation of actin cytoskeleton, autophagy, energy metabolism, and lysosome transport.

Although we identified the immune and inflammation-related pathways markedly increased in cerebral ischemia, we also observed that the pathways regulating synaptic plasticity, neurotransmission, and dendrite organization markedly decreased in response to cerebral I/R, which is consistent with previously reported changes in cerebral I/R. Synaptic dysfunction is one of the pathophysiological events triggered by cerebral I/R, eventually leading to neuronal death (76, 82). Activated microglia and their interaction with other cells can participate in synaptic plasticity (83). Therefore, our findings imply that the activation of the immune inflammatory response triggered by cerebral I/R may trigger synaptic dysfunction and eventually lead to neuron injury. It is quite interesting to test if the activation of the inflammatory responses leads to the loss of synaptic function or if both jointly aggravate neuronal injury after cerebral I/R in the future. In addition, many of the key proteins involved in signal transduction pathways are highly regulated by the phosphorylation (84), and the experimental validation of phosphoproteome for understanding the phosphorylation patterns of some crucial proteins involved in cerebral I/R is required in the future. Thus, integrated phosphoproteome and proteome profiles and the changes in different pathways of cerebral I/R in this study may provide a resource to understand the complicated molecular mechanism of cerebral I/R. Further clarifying the DEGs and related pathways will be helpful for the prevention and therapy of cerebral I/R.

Interestingly, several protease inhibitors, mainly secreted serine protease inhibitors, were significantly upregulated in our proteome and transcriptome profiles. Serine protease inhibitors are closely involved in innate and adaptive immune responses (85). Serpina3n (serine peptidase inhibitor clade A member 3N) has been previously described as a potential marker of reactive astrogliosis. Serpina3n is upregulated in astrocytes and neurons within the ischemic penumbra after stroke and reduces brain damage possibly by interacting with clusterin and inhibiting neuronal apoptosis and neuroinflammation (86, 87). Moreover, our data showed that Serpina3n has not changed in BV2 microglial cells after OGD/R (Fig. 3*F* and supplemental Fig. S4*A*). These results indicated that Serpina3n may regulate immune inflammatory responses in astrocytes or neurons in response to cerebral I/R.

Our transcriptomic analyses in brain tissue also showed that multiple immune inflammatory–related pathways could be activated in the hippocampus after cerebral I/R, including the NF-κB signaling pathway. Previous studies have suggested that inhibiting immune-inflammatory response attenuates ischemia-induced brain injury (88). Intriguingly, our combinatorial analyses of proteome, phosphoproteome, and transcriptome of I/R mouse hippocampi revealed that Anxa2 had a high correlation to inflammation responses during I/R. Previous studies reported that Anxa2 is upregulated in the mouse brain after traumatic spinal cord injury (33, 89). Consistently, we found that Anxa2 is significantly increased after cerebral I/R. However, the most important finding in our study is that Anxa2 was specifically upregulated in microglia cells after cerebral I/R.

To test the hypothesis that Anxa2 might be involved in the progress of cerebral I/R injury in microglia, we also obtained microglia-specific transcriptomic profiles of Anxa2 during OGD/R. Interestingly, we found that Anxa2 knockdown in BV2 microglial cells with OGD/R treatment can reverse some gene expression profiles, especially the inflammation-related ones in BV2 microglial cells. We analyzed DEGs mainly enriched in inflammation-related biological processes and pathways such as NF-κB. Additionally, we found arginine and proline metabolism changes after Anxa2 knockdown with OGD/R treatment. It has been concluded that different polarized types of microglia and their secreted factors affect energy metabolism. iNOS and Arg-1 participate in arginine metabolism, where they compete with each other for arginine metabolism substrates during arginine metabolism. The maintenance of Arg-1 high expression can produce proline or polyamine and NO generation at a low level, which helps with neuroprotection (90). We further demonstrated that Anxa2 knockdown promoted anti-inflammatory phenotype but reduced pro-inflammatory phenotype to exert anti-inflammatory function in microglia under OGD/R conditions. Our study revealed that Anxa2 regulates the release of Arg-1 and iNOS in microglia, suggesting that Anxa2 might affect inflammation by affecting arginine and proline metabolism. However, these possibilities remain to be further investigated in the future. Based on the *in vitro* model, we also found that upregulation of Anxa2 promotes the production of pro-inflammatory cytokines, including TNF-α, IL-1β, and IL-6, as well as cell apoptosis in BV2 microglial cells.

Meanwhile, Anxa2 knockdown also synchronously alleviated the expression and release of multiple pro-inflammatory cytokines, such as TNF-α, IL-1β, and IL-6. Collectively, our study found that Anxa2 was abundantly expressed in microglia after OGD/R and significantly correlated with inflammation. Further research showed that activation of Anxa2 effectively inhibited the inflammatory response of microglia. Conversely, previous studies suggest that Anxa2 deficiency increases pro-inflammatory response after bacterial infection in the lung (91). Another study showed that loss of Anxa2 increased pro-inflammatory response in the Anxa2 KO mouse brain compared with the WT mice after TBI (33). Although this is inconsistent with previous research, our findings and earlier studies suggested that the role of Anxa2 might be diverse in different cell types or pathological conditions.

Previous studies have demonstrated that Anxa2 silencing alleviates the progression of acute pancreatitis, obesity-induced insulin resistance, and pediatric neuroblastoma by inhibiting the NF-κB signaling pathway (72, 92, 93). Furthermore, several studies have revealed that NF-κB plays a crucial role in the microglial phenotype and inflammatory response. Particularly, some stimulus can promote the transcriptional activation of NF-κB and causes neurotoxicity by releasing cytotoxic substances and inflammatory factors (94, 95, 96). NF-κB is a transcription factor widely known to be associated with inflammatory responses involved in ischemic stroke (97). Our study revealed that NF-κB signaling was activated and subsequently increased the production of pro-inflammatory factors in response to OGD/R, which is consistent with the previous studies (98). Our results demonstrated that Anxa2 knockdown significantly reduces nuclear translocation of NF-κB subunit p65, thereby inhibiting OGD/R-induced NF-κB signaling pathway activation. These results imply that NF-κB signaling might be involved in the regulatory mechanism mediated by Anxa2 in cerebral I/R. However, the study has revealed that Anxa2 interacted with the p50 subunit of NF-κB to regulate the NF-κB signaling pathway (71). However, our results demonstrated that OGD/R did not affect the interaction between Anxa2 and the p50 subunit of NF-κB. We also found Anxa2 translocated to the membrane in microglial cells in response to OGD/R. Several studies have suggested that I/R and OGD/R exposure triggered the elevation of intracellular Ca^2+^ concentration (43, 99). While Anxa2 is a member of the family of Ca^2+^-dependent anionic phospholipid-binding proteins, Anxa2 can bind cell surface receptor S100a10, increasing the Ca^2+^ sensitivity of Anxa2 and leading to binding membranes (100). Because the four C-terminus of Anxa2 harbors the binding sites of Ca^2+^, phospholipids, and F-actin, which are necessary for the membrane-associated activities of Anxa2, this domain also directly interacts with several molecules such as heparin and RNA, thus ensuring Anxa2 with more function (101). These indicate OGD/R may induce Anxa2 to translocate to the membrane through Ca^2+^. Therefore, Anxa2 regulates the expression of inflammatory factors and may also be because NF-κB can indirectly affect the expression of these genes by regulating other signaling or transcription factor pathways in response to OGD/R in microglial cells. Future studies should focus on revealing the precise molecular mechanisms of Anxa2 regulating inflammatory response.

Neuronal death is one of the pathological events in the progression of cerebral ischemia (102). Our study demonstrated that microglia specifically the knockdown of Anxa2 provided neuroprotective effects in response to OGD/R. We observed that cleaved caspase-3 expression was significantly increased in neurons subjected to the conditional medium from BV2 cells after OGD/R. However, Anxa2 knockdown in microglia markedly reversed these effects, alleviating neuronal apoptosis or death. Moreover, these data further suggested that Anxa2 deficiency suppresses the production of pro-inflammatory factors induced by microglia activation, thereby preventing subsequent neuronal death and ultimately protecting against cerebral ischemia.

Therefore, Anxa2 may monitor the local environment and regulate inflammatory response after cerebral I/R injury. These findings revealed a novel function of Anxa2 in cerebral I/R injury and indicated that intervening in target Anxa2 expression in microglia may be a potential therapeutic strategy for alleviating the pathological progression of cerebral ischemia.

Although our study revealed an important role of microglial Anxa2 in the protection of neurons against cerebral I/R and provided useful information for the studies of the molecular mechanisms or biomarkers for cerebral I/R injury, especially in immune inflammatory-related pathological processes, how Anxa2 regulates NF-κB transcriptional program needs to be explicitly clarified in the future. Our study only investigated the effect of Anxa2 on the expression and release of pro-inflammatory cytokine *via* the NF-κB signaling pathway in response to OGD/R, and the detailed molecular mechanism will be investigated in the future using Anxa2 conditional and cell-specific KO mice. More cerebral I/R time points could be examined to reveal the dynamic changes in proteins or genes. In addition, further study is necessary to analyze gene expression profiles for more defined brain regions or cell populations during cerebral ischemic stroke using more advanced technologies (*e.g.*, single-cell sequencing). However, our multi-omics profile provides comprehensive insights into which hippocampal genes or proteins participate in cerebral I/R pathology.

**Fig. 8:**
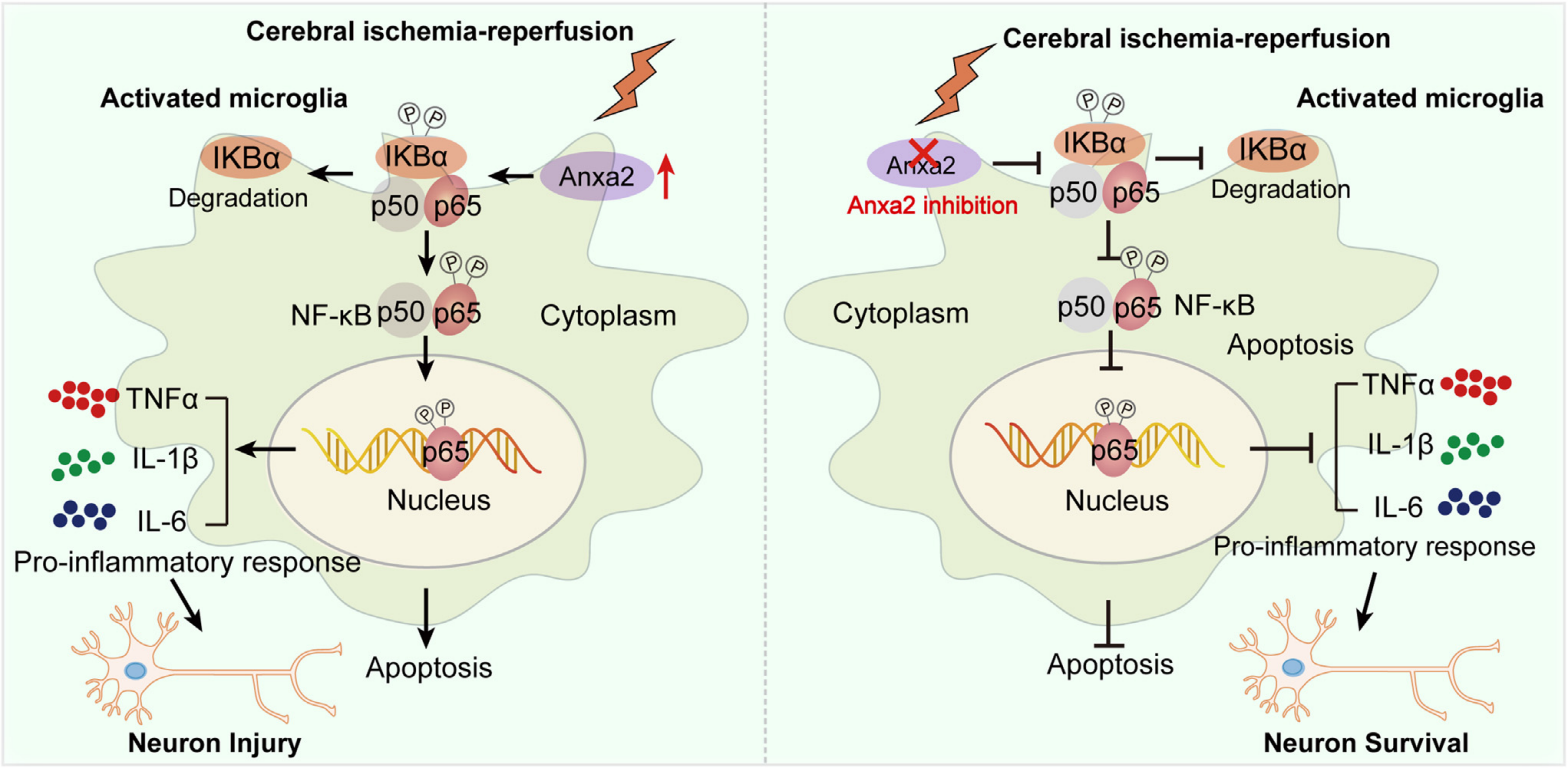
**Graphical illustration of the potential molecular mechanism of the Anxa2 gene in cerebral ischemia-reperfusion injury.** Anxa2 in microglia is activated following ischemia-reperfusion; upregulation of Anxa2 expression can activate the NF-κB signaling pathway through nuclear translocation of NF-κB. Activated Anxa2 promotes the NF-κB pathway, which promotes the transcription activity of NF-κB, thus promoting the production of pro-inflammatory cytokines and cell apoptosis, thereby promoting subsequent neuronal death; Anxa2 suppression can alleviate OGD/R-induced inflammatory response and apoptosis by inhibiting the activation of the NF-κB signaling pathway and the expression of its downstream pro-inflammatory genes, thereby preventing subsequent neuronal death. Anxa2, annexin A2; NF-κB, nuclear factor-kappa B; OGD/R, oxygen-glucose deprivation and reoxygenation.

In summary, we aimed to explore the possible molecular mechanisms underlying cerebral I/R using comprehensive and combined bioinformatics analyses. We identified a key molecule Anxa2 that regulates microglia activation and inflammation by mediating downstream NF-κB signaling pathway and subsequently modulates neuronal outcome with cerebral I/R (Fig. 8). We found that Anxa2 deficiency attenuates the inflammation response induced by microglia and protects neuronal death. These findings expand our understanding of the biological functions of microglia after cerebral I/R. Thus, our study suggested that targeting the Anxa2-related inflammatory pathway or inflammation-related neuronal damage might offer unique insights into ischemic stroke's pathological processes and help establish effective therapeutic strategies.

## Data Availability

The mass spectrometry proteomics and phosphoproteomic data have been deposited in the ProteomeXchange Consortium *via* the PRIDE (103) partner repository with the dataset identifiers PXD033938 and PXD033851. The annotated MS/MS spectra can be accessed using the respective search key “eihx83br9x” and “xhgtguvnys” in https://msviewer.ucsf.edu/prospector/cgi-bin/msform.cgi?form=msviewer.

The RNA-seq data for mouse brain tissue and BV2 cells have been deposited in Gene Expression Omnibus (GEO) under the accession numbers GSE202391 and GSE223540. All data needed to evaluate the conclusions in the paper are present in the paper and/or the Supplemental data. Additional data related to this paper may be requested from the authors.

## Ethics Approval and Consent to Participate

All animal procedures were approved by the Institute of Animal Care Committee of Hunan University (No. SYXK [Xiang] 2023-0010) and were performed in accordance with institutional and national guidelines and regulations. Efforts were made to minimize animal suffering.

## Supplemental data

This article contains supplemental data (71).

## Conflict of interests

The authors declare no competing interests.

## References

[1] Jia J., Deng J., Jin H., Yang J., Nan D., Yu Z. (2023). Effect of Dl-3-n-butylphthalide on mitochondrial Cox7c in models of cerebral ischemia/reperfusion injury. Front. Pharmacol..

[2] Liu J., Luo Q., Ke J., Zhang D., Xu Y., Liao W. (2023). Enriched environment attenuates ferroptosis after cerebral ischemia/reperfusion injury *via* the HIF-1alpha-ACSL4 pathway. Oxid. Med. Cell Longev..

[3] Mendelson S.J., Prabhakaran S. (2021). Diagnosis and management of transient ischemic attack and acute ischemic stroke: a review. JAMA.

[4] Yu T.F., Wang K., Yin L., Li W.Z., Li C.P., Zhang W. (2023). A molecular probe carrying anti-tropomyosin 4 for early diagnosis of cerebral ischemia/reperfusion injury. Neural Regen. Res..

[5] Tsao C.W., Aday A.W., Almarzooq Z.I., Anderson C.A.M., Arora P., Avery C.L. (2023). Heart disease and stroke statistics-2023 update: a report from the American heart association. Circulation.

[6] Hu M., Huang J., Chen L., Sun X.R., Yao Z.M., Tong X.H. (2023). Upregulation of CDGSH iron sulfur domain 2 attenuates cerebral ischemia/reperfusion injury. Neural Regen. Res..

[7] Liu Y., Chen S., Liu S., Wallace K.L., Zille M., Zhang J. (2023). T-cell receptor signaling modulated by the co-receptors: potential targets for stroke treatment. Pharmacol. Res..

[8] Xu P., Kong L., Tao C., Zhu Y., Cheng J., Li W. (2023). Elabela-APJ axis attenuates cerebral ischemia/reperfusion injury by inhibiting neuronal ferroptosis. Free Radic. Biol. Med..

[9] Zhang L., Han Y., Wu X., Chen B., Liu S., Huang J. (2023). Research progress on the mechanism of curcumin in cerebral ischemia/reperfusion injury: a narrative review. Apoptosis.

[10] Soares R.O.S., Losada D.M., Jordani M.C., Evora P., Castro E.S.O. (2019). Ischemia/reperfusion injury revisited: an overview of the latest pharmacological strategies. Int. J. Mol. Sci..

[11] Zhou F., Wang Y.K., Zhang C.G., Wu B.Y. (2021). miR-19a/b-3p promotes inflammation during cerebral ischemia/reperfusion injury *via* SIRT1/FoxO3/SPHK1 pathway. J. Neuroinflammation.

[12] Shi K., Tian D.C., Li Z.G., Ducruet A.F., Lawton M.T., Shi F.D. (2019). Global brain inflammation in stroke. Lancet Neurol..

[13] Li C., Zhao B., Lin C., Gong Z., An X. (2019). TREM2 inhibits inflammatory responses in mouse microglia by suppressing the PI3K/NF-κB signaling. Cell Biol. Inter..

[14] Ma Y., Wang J., Wang Y., Yang G.Y. (2017). The biphasic function of microglia in ischemic stroke. Prog. Neurobiol..

[15] Voet S., Prinz M., van Loo G. (2019). Microglia in central nervous system inflammation and multiple sclerosis pathology. Trends Mol. Med..

[16] Jurcau A., Simion A. (2021). Neuroinflammation in cerebral ischemia and ischemia/reperfusion injuries: from pathophysiology to therapeutic strategies. Int. J. Mol. Sci..

[17] Zhang B., Wei Y.Z., Wang G.Q., Li D.D., Shi J.S., Zhang F. (2018). Targeting MAPK pathways by naringenin modulates microglia M1/M2 polarization in lipopolysaccharide-stimulated cultures. Front. Cell Neurosci..

[18] Gao H., Ju F., Ti R., Zhang Y., Zhang S. (2022). Differential regulation of microglial activation in response to different degree of ischemia. Front. Immunol..

[19] Wu F., Luo T., Mei Y., Liu H., Dong J., Fang Y. (2018). Simvastatin alters M1/M2 polarization of murine BV2 microglia *via* Notch signaling. J. Neuroimmunol..

[20] Muhammad S., Chaudhry S.R., Kahlert U.D., Niemela M., Hanggi D. (2021). Brain immune interactions-novel emerging options to treat acute ischemic brain injury. Cells.

[21] Iadecola C., Buckwalter M.S., Anrather J. (2020). Immune responses to stroke: mechanisms, modulation, and therapeutic potential. J. Clin. Invest..

[22] Jayaraj R.L., Azimullah S., Beiram R., Jalal F.Y., Rosenberg G.A. (2019). Neuroinflammation: friend and foe for ischemic stroke. J. Neuroinflammation.

[23] Simmons L.J., Surles-Zeigler M.C., Li Y., Ford G.D., Newman G.D., Ford B.D. (2016). Regulation of inflammatory responses by neuregulin-1 in brain ischemia and microglial cells *in vitro* involves the NF-kappa B pathway. J. Neuroinflammation.

[24] Dallacasagrande V., Hajjar K.A. (2020). Annexin A2 in inflammation and host defense. Cells.

[25] Huang B., Deora A.B., He K.L., Chen K., Sui G., Jacovina A.T. (2011). Hypoxia-inducible factor-1 drives annexin A2 system-mediated perivascular fibrin clearance in oxygen-induced retinopathy in mice. Blood.

[26] Jiang S., Xu Y. (2019). Annexin A2 upregulation protects human retinal endothelial cells from oxygen-glucose deprivation injury by activating autophagy. Exp. Therap. Med..

[27] Li W., Chen Z., Yuan J., Yu Z., Cheng C., Zhao Q. (2019). Annexin A2 is a Robo4 ligand that modulates ARF6 activation-associated cerebral trans-endothelial permeability. J. Cereb. Blood Flow Metab..

[28] Fan X., Jiang Y., Yu Z., Liu Q., Guo S., Sun X. (2017). Annexin A2 plus low-dose tissue plasminogen activator combination attenuates cerebrovascular dysfunction after focal embolic stroke of rats. Transl. Stroke Res..

[29] Lin H., Li W., Shen Z., Bei Y., Wei T., Yu Z. (2023). Annexin A2 promotes angiogenesis after ischemic stroke *via* annexin A2 receptor - AKT/ERK pathways. Neurosci. Lett..

[30] Scharf B., Clement C.C., Wu X.X., Morozova K., Zanolini D., Follenzi A. (2012). Annexin A2 binds to endosomes following organelle destabilization by particulate wear debris. Nat. Commun..

[31] Zhang S., Yu M., Guo Q., Li R., Li G., Tan S. (2015). Annexin A2 binds to endosomes and negatively regulates TLR4-triggered inflammatory responses *via* the TRAM-TRIF pathway. Sci. Rep..

[32] He S., Li X., Li R., Fang L., Sun L., Wang Y. (2016). Annexin A2 modulates ROS and impacts inflammatory response *via* IL-17 signaling in polymicrobial sepsis mice. PLoS Pathog..

[33] Liu N., Jiang Y., Chung J.Y., Li Y., Yu Z., Kim J.W. (2019). Annexin a2 deficiency exacerbates neuroinflammation and long-term neurological deficits after traumatic brain injury in mice. Inter. J. Mol. Sc..

[34] Liu N., Han J., Li Y., Jiang Y., Shi S.X., Lok J. (2021). Recombinant annexin A2 inhibits peripheral leukocyte activation and brain infiltration after traumatic brain injury. J. Neuroinflammation.

[35] Iadecola C., Anrather J. (2011). The immunology of stroke: from mechanisms to translation. Nat. Med..

[36] Zeng J., Wang Y., Luo Z., Chang L.C., Yoo J.S., Yan H. (2019). TRIM9-Mediated resolution of neuroinflammation confers neuroprotection upon ischemic stroke in mice. Cell Rep..

[37] Uluc K., Miranpuri A., Kujoth G.C., Akture E., Baskaya M.K. (2011). Focal cerebral ischemia model by endovascular suture occlusion of the middle cerebral artery in the rat. J. Vis. Exp..

[38] Jiang W., Zhang P., Yang P., Kang N., Liu J., Aihemaiti Y. (2022). Phosphoproteome analysis identifies a synaptotagmin-1-associated complex involved in ischemic neuron injury. Mol. Cell Proteomics.

[39] Zhang X., Zhou Q., Zou W., Hu X. (2017). Molecular mechanisms of developmental toxicity induced by graphene oxide at predicted environmental concentrations. Environ. Sci. Technol..

[40] Tang Q., Liu M., Liu Y., Hwang R.D., Zhang T., Wang J. (2021). NDST3 deacetylates α-tubulin and suppresses V-ATPase assembly and lysosomal acidification. EMBO J..

[41] Bi M., Gladbach A., van Eersel J., Ittner A., Przybyla M., van Hummel A. (2017). Tau exacerbates excitotoxic brain damage in an animal model of stroke. Nat. Commun..

[42] Zhao X., Liao Y., Morgan S., Mathur R., Feustel P., Mazurkiewicz J. (2018). Noninflammatory changes of microglia are sufficient to cause epilepsy. Cell Rep..

[43] Sugiyama A., Shimizu Y., Okada M., Otani K., Yamawaki H. (2021). Preventive effect of canstatin against ventricular arrhythmia induced by ischemia/reperfusion injury: a pilot study. Int. J. Mol. Sci..

[44] Xu X., Gao W., Li L., Hao J., Yang B., Wang T. (2021). Annexin A1 protects against cerebral ischemia-reperfusion injury by modulating microglia/macrophage polarization *via* FPR2/ALX-dependent AMPK-mTOR pathway. J. Neuroinflammation.

[45] Wang T., Tian X., Kim H.B., Jang Y., Huang Z., Na C.H. (2022). Intracellular energy controls dynamics of stress-induced ribonucleoprotein granules. Nat. Commun..

[46] Tian M., Yang M., Li Z., Wang Y., Chen W., Yang L. (2019). Fluoxetine suppresses inflammatory reaction in microglia under OGD/R challenge *via* modulation of NF-kappaB signaling. Biosci. Rep..

[47] Wang T., Liu H., Itoh K., Oh S., Zhao L., Murata D. (2021). C9orf72 regulates energy homeostasis by stabilizing mitochondrial complex I assembly. Cell Metab..

[48] Rosner M., Schipany K., Hengstschlager M. (2013). Merging high-quality biochemical fractionation with a refined flow cytometry approach to monitor nucleocytoplasmic protein expression throughout the unperturbed mammalian cell cycle. Nat. Protoc..

[49] Fanunza E., Frau A., Sgarbanti M., Orsatti R., Corona A., Tramontano E. (2018). Development and validation of a novel dual luciferase reporter gene assay to quantify ebola virus VP24 inhibition of IFN signaling. Viruses.

[50] Wang G., Wang T., Zhang Y., Li F., Yu B., Kou J. (2019). Schizandrin protects against OGD/R-Induced neuronal injury by suppressing autophagy: involvement of the AMPK/mTOR pathway. Molecules.

[51] Doyle K.P., Yang T., Lessov N.S., Ciesielski T.M., Stevens S.L., Simon R.P. (2008). Nasal administration of osteopontin peptide mimetics confers neuroprotection in stroke. J. Cereb. Blood Flow Metab..

[52] Lin E.Y., Xi W., Aggarwal N., Shinohara M.L. (2022). Osteopontin (OPN)/SPP1: from its biochemistry to biological functions in the innate immune system and the central nervous system (CNS). Int. Immunol..

[53] Wolak T., Kim H., Ren Y., Kim J., Vaziri N.D., Nicholas S.B. (2009). Osteopontin modulates angiotensin II-induced inflammation, oxidative stress, and fibrosis of the kidney. Kidney Int..

[54] Heilmann K., Hoffmann U., Witte E., Loddenkemper C., Sina C., Schreiber S. (2009). Osteopontin as two-sided mediator of intestinal inflammation. J. Cell Mol. Med..

[55] Lok Z.S.Y., Lyle A.N. (2019). Osteopontin in vascular disease. Arterioscler. Thromb. Vasc. Biol..

[56] Carbone F., Vuilleumier N., Burger F., Roversi G., Tamborino C., Casetta I. (2015). Serum osteopontin levels are upregulated and predict disability after an ischaemic stroke. Eur. J. Clin. Invest..

[57] Nie Q.Q., Zheng Z.Q., Liao J., Li Y.C., Chen Y.T., Wang T.Y. (2022). SPP1/AnxA1/TIMP1 as essential genes regulate the inflammatory response in the acute phase of cerebral ischemia-reperfusion in rats. J. Inflamm. Res..

[58] Zhu Q., Luo X., Zhang J., Liu Y., Luo H., Huang Q. (2017). Osteopontin as a potential therapeutic target for ischemic stroke. Curr. Drug Deliv..

[59] Christensen B., Kazanecki C.C., Petersen T.E., Rittling S.R., Denhardt D.T., Sorensen E.S. (2007). Cell type-specific post-translational modifications of mouse osteopontin are associated with different adhesive properties. J. Biol. Chem..

[60] Mateos B., Holzinger J., Conrad-Billroth C., Platzer G., Zerko S., Sealey-Cardona M. (2021). Hyperphosphorylation of human osteopontin and its impact on structural dynamics and molecular recognition. Biochemistry.

[61] Christensen B., Nielsen M.S., Haselmann K.F., Petersen T.E., Sorensen E.S. (2005). Post-translationally modified residues of native human osteopontin are located in clusters: identification of 36 phosphorylation and five O-glycosylation sites and their biological implications. Biochem. J..

[62] Gundimeda U., McNeill T.H., Elhiani A.A., Schiffman J.E., Hinton D.R., Gopalakrishna R. (2012). Green tea polyphenols precondition against cell death induced by oxygen-glucose deprivation *via* stimulation of laminin receptor, generation of reactive oxygen species, and activation of protein kinase Cepsilon. J. Biol. Chem..

[63] Zhang Y., Lian L., Fu R., Liu J., Shan X., Jin Y. (2022). Microglia: the hub of intercellular communication in ischemic stroke. Front. Cell Neurosci..

[64] Weinstein J.R., Koerner I.P., Moller T. (2010). Microglia in ischemic brain injury. Future Neurol..

[65] Liao Y., Cheng J., Kong X., Li S., Li X., Zhang M. (2020). HDAC3 inhibition ameliorates ischemia/reperfusion-induced brain injury by regulating the microglial cGAS-STING pathway. Theranostics.

[66] Thiel C., Osborn M., Gerke V. (1992). The tight association of the tyrosine kinase substrate annexin II with the submembranous cytoskeleton depends on intact p11- and Ca(2+)-binding sites. J. Cell Sci..

[67] He T., Li W., Song Y., Li Z., Tang Y., Zhang Z. (2020). Sestrin2 regulates microglia polarization through mTOR-mediated autophagic flux to attenuate inflammation during experimental brain ischemia. J. Neuroinflammation.

[68] He D., Fu S., Ye B., Wang H., He Y., Li Z. (2023). Activation of HCA2 regulates microglial responses to alleviate neurodegeneration in LPS-induced *in vivo* and *in vitro* models. J. Neuroinflammation.

[69] Singh R., Letai A., Sarosiek K. (2019). Regulation of apoptosis in health and disease: the balancing act of BCL-2 family proteins. Nat. Rev. Mol. Cell Biol..

[70] Jover-Mengual T., Hwang J.Y., Byun H.R., Court-Vazquez B.L., Centeno J.M., Burguete M.C. (2021). The role of NF-kappaB triggered inflammation in cerebral ischemia. Front. Cell Neurosci..

[71] Jung H., Kim J.S., Kim W.K., Oh K.J., Kim J.M., Lee H.J. (2015). Intracellular annexin A2 regulates Nf-kB signaling by binding to the p50 subunit: implications for gemcitabine resistance in pancreatic cancer. Cell Death Dis..

[72] Wang Y., Chen K., Cai Y., Cai Y., Yuan X., Wang L. (2017). Annexin A2 could enhance multidrug resistance by regulating NF-κB signaling pathway in pediatric neuroblastoma. J. Exp. Clin. Cancer Res..

[73] Mankan A.K., Lawless M.W., Gray S.G., Kelleher D., McManus R. (2009). NF-kappaB regulation: the nuclear response. J. Cell Mol. Med..

[74] Cai Y., Zhang Y., Ke X., Guo Y., Yao C., Tang N. (2019). Transcriptome sequencing unravels potential biomarkers at different stages of cerebral ischemic stroke. Front. Genet..

[75] Duan G., Walther D. (2015). The roles of post-translational modifications in the context of protein interaction networks. PLoS Comput. Biol..

[76] Wen M., Jin Y., Zhang H., Sun X., Kuai Y., Tan W. (2019). Proteomic analysis of rat cerebral cortex in the subacute to long-term phases of focal cerebral ischemia-reperfusion injury. J. Proteome Res..

[77] Agarwal A., Park S., Ha S., Kwon J.S., Khan M.R., Kang B.G. (2020). Quantitative mass spectrometric analysis of the mouse cerebral cortex after ischemic stroke. PLoS One.

[78] Simats A., Ramiro L., Garcia-Berrocoso T., Brianso F., Gonzalo R., Martin L. (2020). A mouse brain-based multi-omics integrative approach reveals potential blood biomarkers for ischemic stroke. Mol. Cell Proteomics.

[79] Li L., Dong L., Xiao Z., He W., Zhao J., Pan H. (2020). Integrated analysis of the proteome and transcriptome in a MCAO mouse model revealed the molecular landscape during stroke progression. J. Adv. Res..

[80] Tajiri S., Oyadomari S., Yano S., Morioka M., Gotoh T., Hamada J.I. (2004). Ischemia-induced neuronal cell death is mediated by the endoplasmic reticulum stress pathway involving CHOP. Cell Death Differ..

[81] Lana D., Ugolini F., Giovannini M.G. (2020). An overview on the differential interplay among neurons-astrocytes-microglia in CA1 and CA3 Hippocampus in hypoxia/ischemia. Front. Cell Neurosci..

[82] Wang C., Liu M., Pan Y., Bai B., Chen J. (2017). Global gene expression profile of cerebral ischemia-reperfusion injury in rat MCAO model. Oncotarget.

[83] Wu Y., Dissing-Olesen L., MacVicar B.A., Stevens B. (2015). Microglia: dynamic mediators of synapse development and plasticity. Trends Immunol..

[84] Ramazi S., Zahiri J. (2021). Posttranslational modifications in proteins: resources, tools and prediction methods. Database (Oxford).

[85] Li Y., Wang C., Li T., Ma L., Fan F., Jin Y. (2019). The whole transcriptome and proteome changes in the early stage of myocardial infarction. Cell Death Discov..

[86] Xi Y., Liu M., Xu S., Hong H., Chen M., Tian L. (2019). Inhibition of SERPINA3N-dependent neuroinflammation is essential for melatonin to ameliorate trimethyltin chloride–induced neurotoxicity. J. Pineal Res..

[87] Zhang Y., Chen Q., Chen D., Zhao W., Wang H., Yang M. (2022). SerpinA3N attenuates ischemic stroke injury by reducing apoptosis and neuroinflammation. CNS Neurosci. Therap..

[88] Franco E.C.S., Cardoso M.M., Gouvêia A., Pereira A., Gomes-Leal W. (2012). Modulation of microglial activation enhances neuroprotection and functional recovery derived from bone marrow mononuclear cell transplantation after cortical ischemia. Neurosci. Res..

[89] Chen J., Cui Z., Yang S., Wu C., Li W., Bao G. (2017). The upregulation of annexin A2 after spinal cord injury in rats may have implication for astrocyte proliferation. Neuropeptides.

[90] Aaboe Jorgensen M., Ugel S., Linder Hubbe M., Carretta M., Perez-Penco M., Weis-Banke S.E. (2021). Arginase 1-based immune modulatory vaccines induce anticancer immunity and synergize with anti-PD-1 checkpoint blockade. Cancer Immunol. Res..

[91] Li R., Tan S., Yu M., Jundt M.C., Zhang S., Wu M. (2015). Annexin A2 regulates autophagy in Pseudomonas aeruginosa infection through the akt1-mTOR-ULK1/2 signaling pathway. J. Immunol..

[92] Wang Y., Cheng Y.S., Yin X.Q., Yu G., Jia B.L. (2019). Anxa2 gene silencing attenuates obesity-induced insulin resistance by suppressing the NF-kappaB signaling pathway. Am. J. Physiol. Cell Physiol..

[93] Tang G., Yu C., Xiang K., Gao M., Liu Z., Yang B. (2022). Inhibition of ANXA2 regulated by SRF attenuates the development of severe acute pancreatitis by inhibiting the NF-kappaB signaling pathway. Inflamm. Res..

[94] Lang G.P., Li C., Han Y.Y. (2021). Rutin pretreatment promotes microglial M1 to M2 phenotype polarization. Neural Regen. Res..

[95] Taetzsch T., Levesque S., McGraw C., Brookins S., Luqa R., Bonini M.G. (2015). Redox regulation of NF-kappaB p50 and M1 polarization in microglia. Glia.

[96] Chen J., Sun Z., Jin M., Tu Y., Wang S., Yang X. (2017). Inhibition of AGEs/RAGE/Rho/ROCK pathway suppresses non-specific neuroinflammation by regulating BV2 microglial M1/M2 polarization through the NF-kappaB pathway. J. Neuroimmunol..

[97] Zaghloul N., Kurepa D., Bader M.Y., Nagy N., Ahmed M.N. (2020). Prophylactic inhibition of NF-κB expression in microglia leads to attenuation of hypoxic ischemic injury of the immature brain. J. Neuroinflammation.

[98] Ding B., Lin C., Liu Q., He Y., Ruganzu J.B., Jin H. (2020). Tanshinone IIA attenuates neuroinflammation *via* inhibiting RAGE/NF-kappaB signaling pathway *in vivo* and *in vitro*. J. Neuroinflammation.

[99] Wu L.Y., Yu X.L., Feng L.Y. (2015). Connexin 43 stabilizes astrocytes in a stroke-like milieu to facilitate neuronal recovery. Acta Pharmacol. Sin..

[100] Liu Y., Myrvang H.K., Dekker L.V. (2015). Annexin A2 complexes with S100 proteins: structure, function and pharmacological manipulation. Br. J. Pharmacol..

[101] Xu X.H., Pan W., Kang L.H., Feng H., Song Y.Q. (2015). Association of Annexin A2 with cancer development (review). Oncol. Rep..

[102] Li X., Zhao Y., Xia Q., Zheng L., Liu L., Zhao B. (2016). Nuclear translocation of annexin 1 following oxygen-glucose deprivation-reperfusion induces apoptosis by regulating Bid expression *via* p53 binding. Cell Death Dis..

[103] Perez-Riverol Y., Bai J., Bandla C., Garcia-Seisdedos D., Hewapathirana S., Kamatchinathan S. (2022). The PRIDE database resources in 2022: a hub for mass spectrometry-based proteomics evidences. Nucleic Acids Res..

