# Supplementary material for: Multi-Omics Profiling Identifies Microglial Annexin A2 as a Key Mediator of NF-κB Pro-inflammatory Signaling in Ischemic Reperfusion Injury
Source: Mol Cell Proteomics. 2024 Jan 20;23(2):100723. doi: 10.1016/j.mcpro.2024.100723 (PMC10879806; doi:10.1016/j.mcpro.2024.100723)
Supplement: Supplemental data [file mmc12.docx]

**Multi-omics profiling identifies microglial Annexin A2 as a key mediator of**

**NF-κB pro-inflammatory signaling in ischemic reperfusion injury**

**Supplementary Tables**

**Supplemental Table S1.** The differentially expressed proteins of I1.5R24 vs sham in mice.

**Supplemental Table S2.** The differentially expressed phosphoproteins and phosphosites of I1.5R24 vs sham in mice.

**Supplemental Table S3.** Biological process and KEGG pathway for differentially expressed proteins in I1.5R24 vs sham.

**Supplemental Table S4.** Biological process and KEGG pathway for differentially expressed phosphoproteins in I1.5R24 vs sham.

**Supplemental Table S5.** Immune-related proteins and phosphoproteins in proteome and phosphoproteome.

**Supplemental Table S6.** RNA-seq identified differentially expressed genes of I1.5R24 vs sham in mice.

**Supplemental Table S7.** Biological process and KEGG pathway for upregulated genes in I1.5R24 vs sham.

**Supplemental Table S8.** The common DEPs and DEGs of I1.5R24 vs sham in mice.

**Supplemental Tables S9.** RNA-seq identified differentially expressed genes in shCtrl-OGDR vs shctrl-CTRL, shAnxa2-OGDR vs shCtrl-OGDR.

**Supplemental Table S10.** Biological process and KEGG pathway for upregulated genes in shCtrl-OGDR vs shCtrl-CTRL and downregulated genes in shAnxa2-OGDR vs shCtrl-OGDR.

**Supplemental Table S11.** Exact P value of statistics of all panels.

**Supplemental Table S12.** Primer sequences of qRT-PCR for this study.

**Supplementary Figures**

**
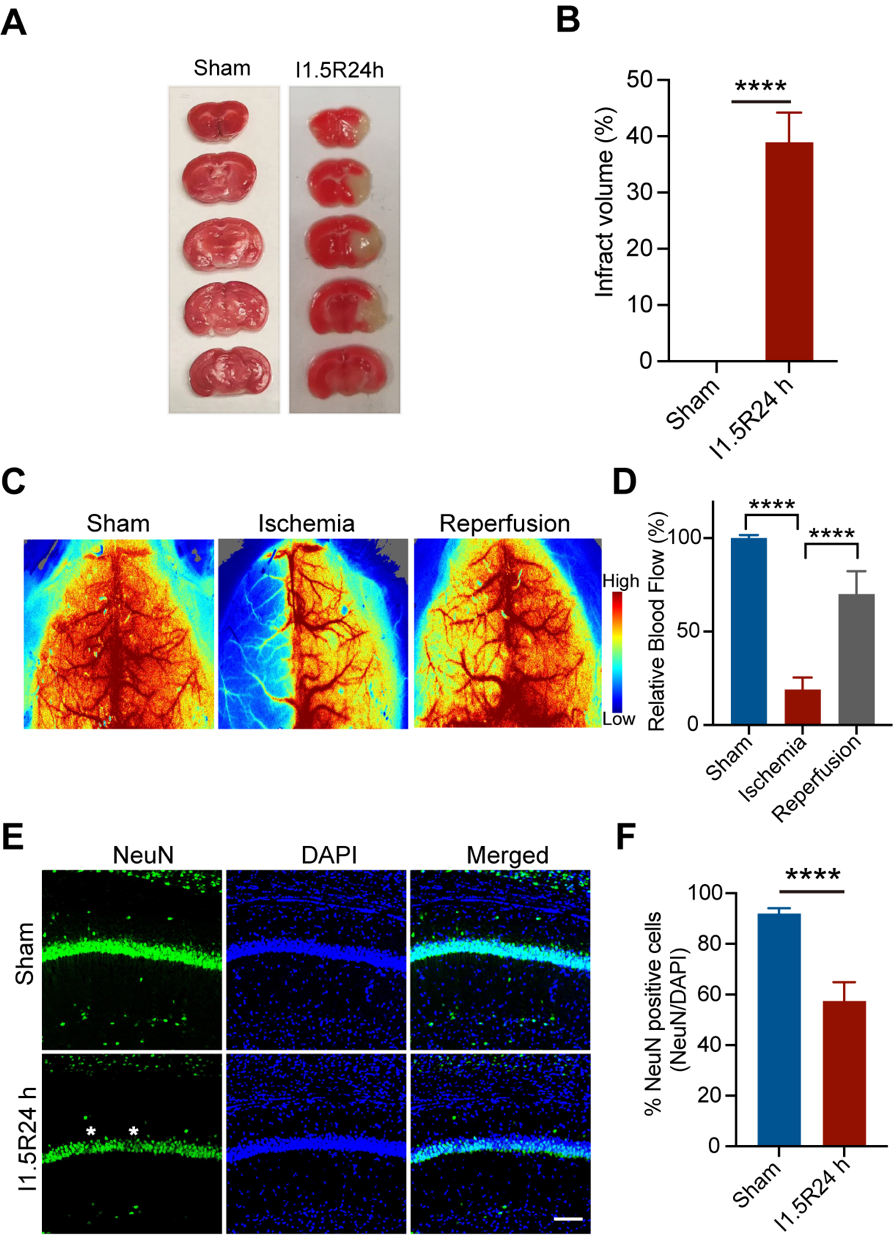
**

**Supplemental Figure S1. Neuronal loss of hippocampal CA1 region after cerebral I/R. (A)** Representative TTC staining images at 24 h after reperfusion. The infarct portion appeared white and the non-infract portion appeared red. **(B)** Quantitative analysis of the infarct volume. **(C)** A Laser Speckle Imaging System monitored cerebral blood flow during sham or MCAO and reperfusion surgery.

**(D)** Quantitative analysis of cerebral blood flow from sham group or MCAO and reperfusion group (n=6). **(E)** Representative NeuN immunoreactive in the hippocampal CA1 regions neuron of the sham group and I/R group. Scale bar, 100 μm. **(F)** Quantitative analysis of the number of NeuN positive neurons in the hippocampal CA1 regions. Data were presented as mean ± SEM. Statistical significance was determined by unpaired student’s t-test for comparisons between two groups. **** *p* < 0.0001.


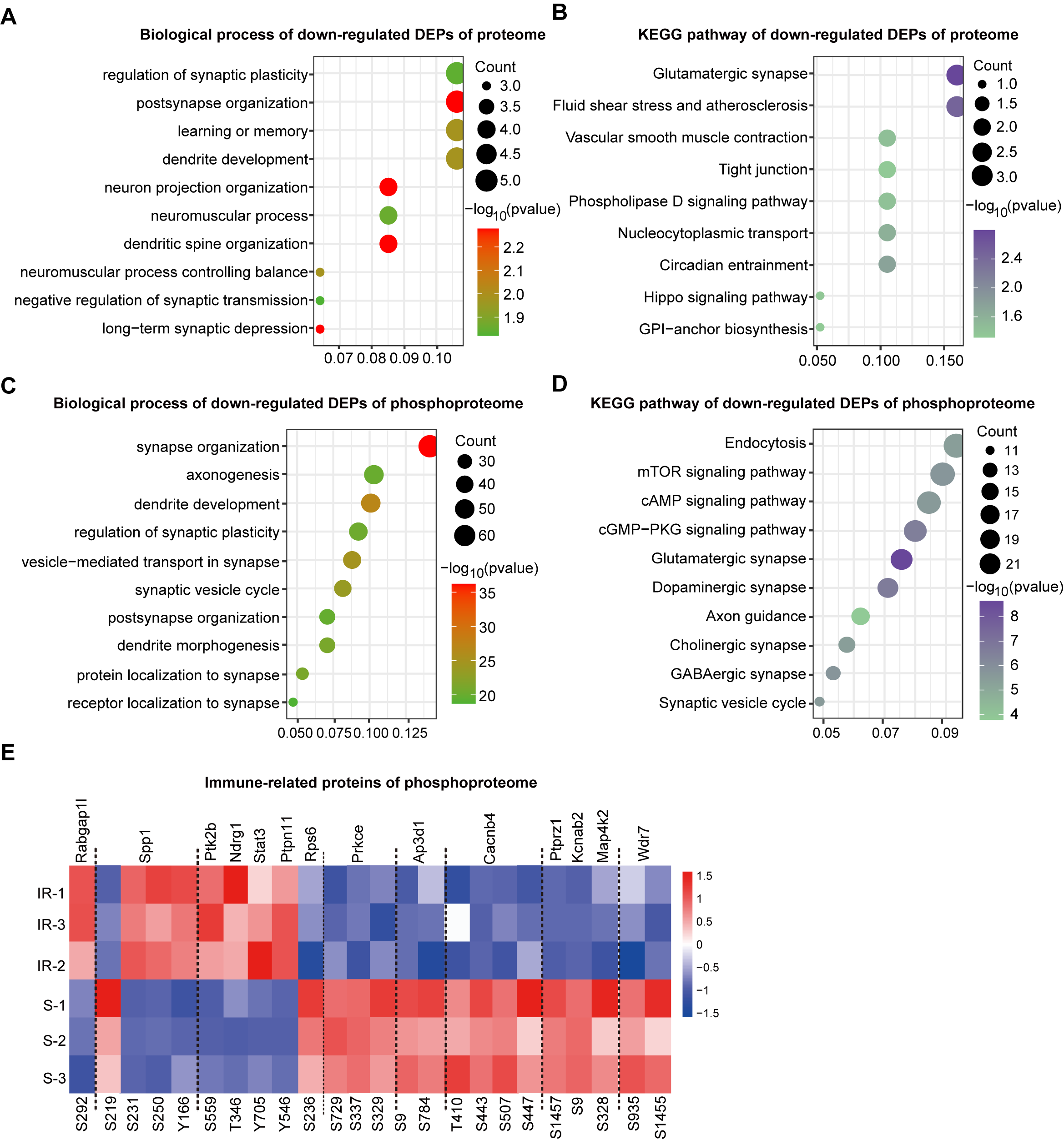


**Supplemental Figure S2. Proteome and phosphoproteome analysis of hippocampus in a mouse model of cerebral ischemia-reperfusion. (A and** **B)** Biological process (A) and KEGG pathway (B) analyses of down-regulated DEPs after I/R in proteome (p-value < 0.05). **(C and D)** Biological process (C) and KEGG pathway (D) analysis of down-regulated DEPs after I/R in phosphoproteome (p-value < 0.05). **(E)** Cluster analysis of the immune inflammatory-related proteins significantly altered after I/R in phosphoproteome. The column on the right, the row listed the replicate samples and the names of immune-related proteins (Upper) and the corresponding phosphorylation sites (Lower), respectively. Red and blue colors represent upregulated and downregulated genes, respectively.


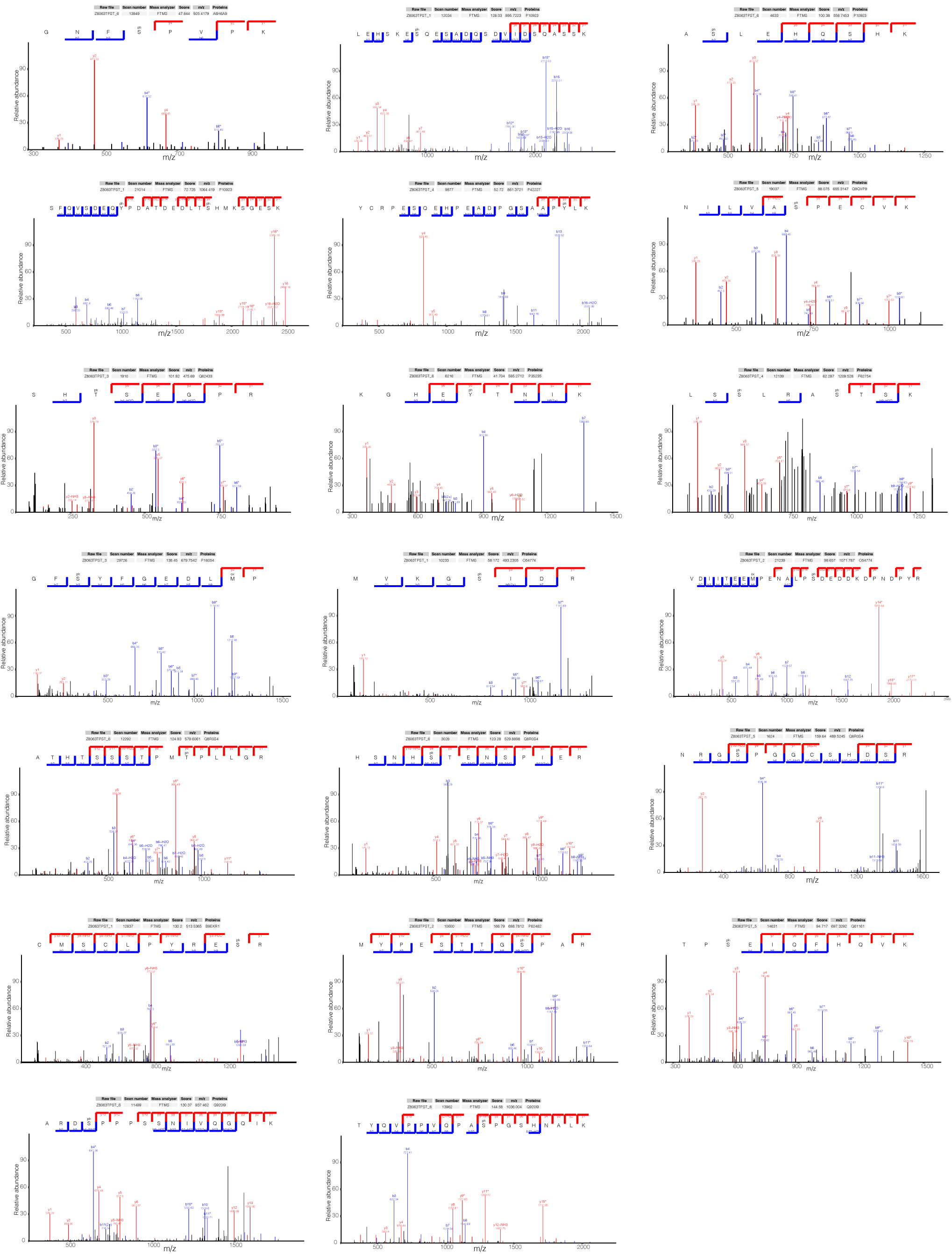


**Supplemental Figure S3.** Mass spectrometry spectrum of the immune-related phosphorylated sites.

**
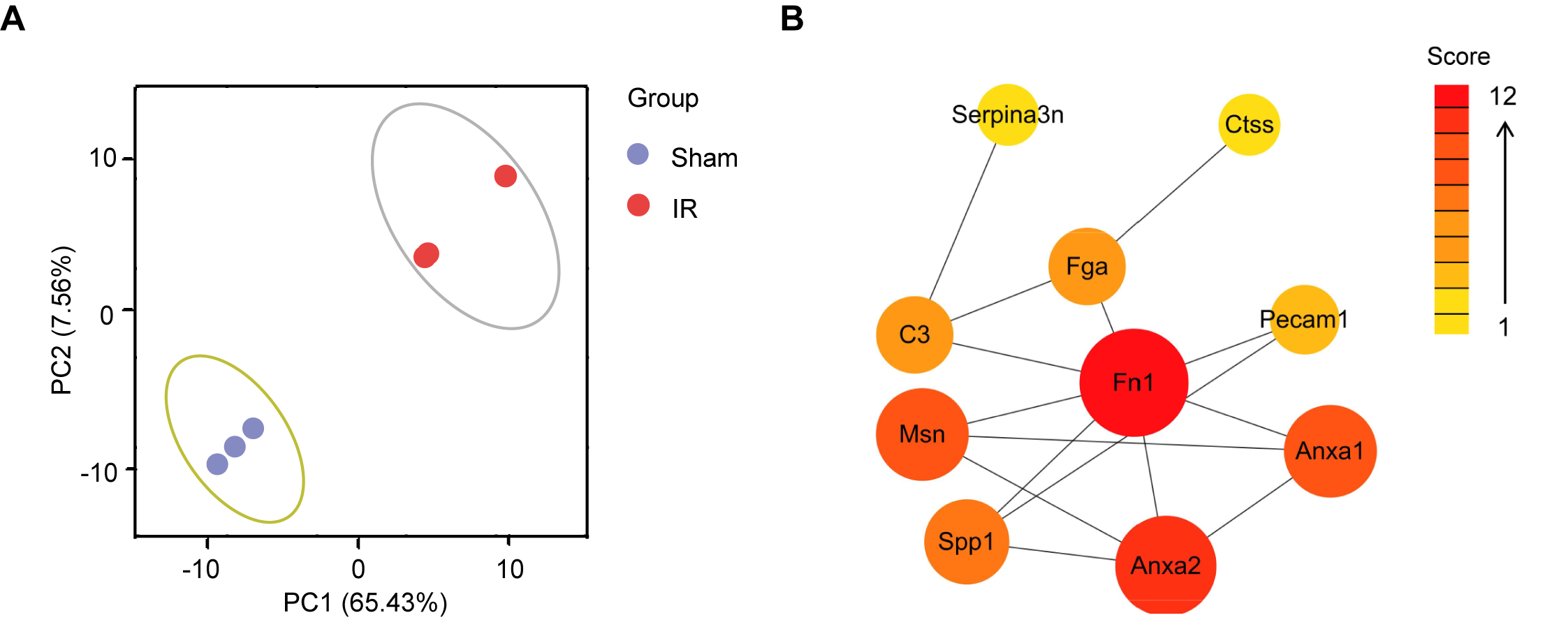
**

**Supplemental Figure S4. Transcriptome analysis of hippocampus in a mouse model of cerebral ischemia-reperfusion showing the important role of Anexa2 in the PPI network. (A)** Principal component analysis (PCA) for three biological replicates of sham and I/R groups in transcriptome profile. Purple and red-filled circles indicate three biological replicates of sham and I/R groups, respectively. **(B)** The PPI network of 11 overlapped genes between transcriptome and proteome is visualized by cytohubba plug-in (the disconnected gene is deleted in the network). The nodes with larger scores displayed in bigger sizes and redder colors indicate more important role of genes in the network.


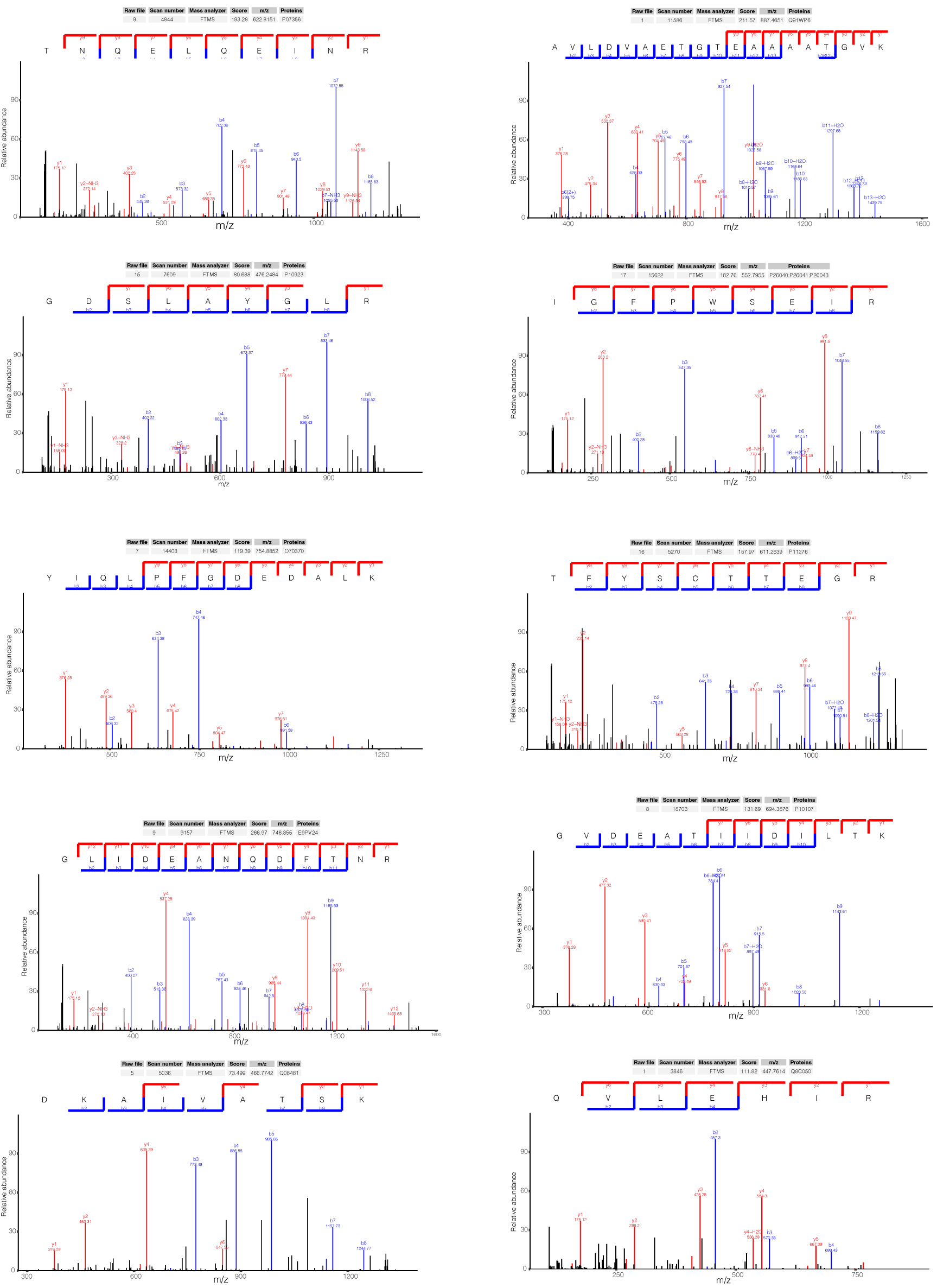


**Supplemental Figure S5.** Mass spectrometry spectrum of the immune-related proteins.


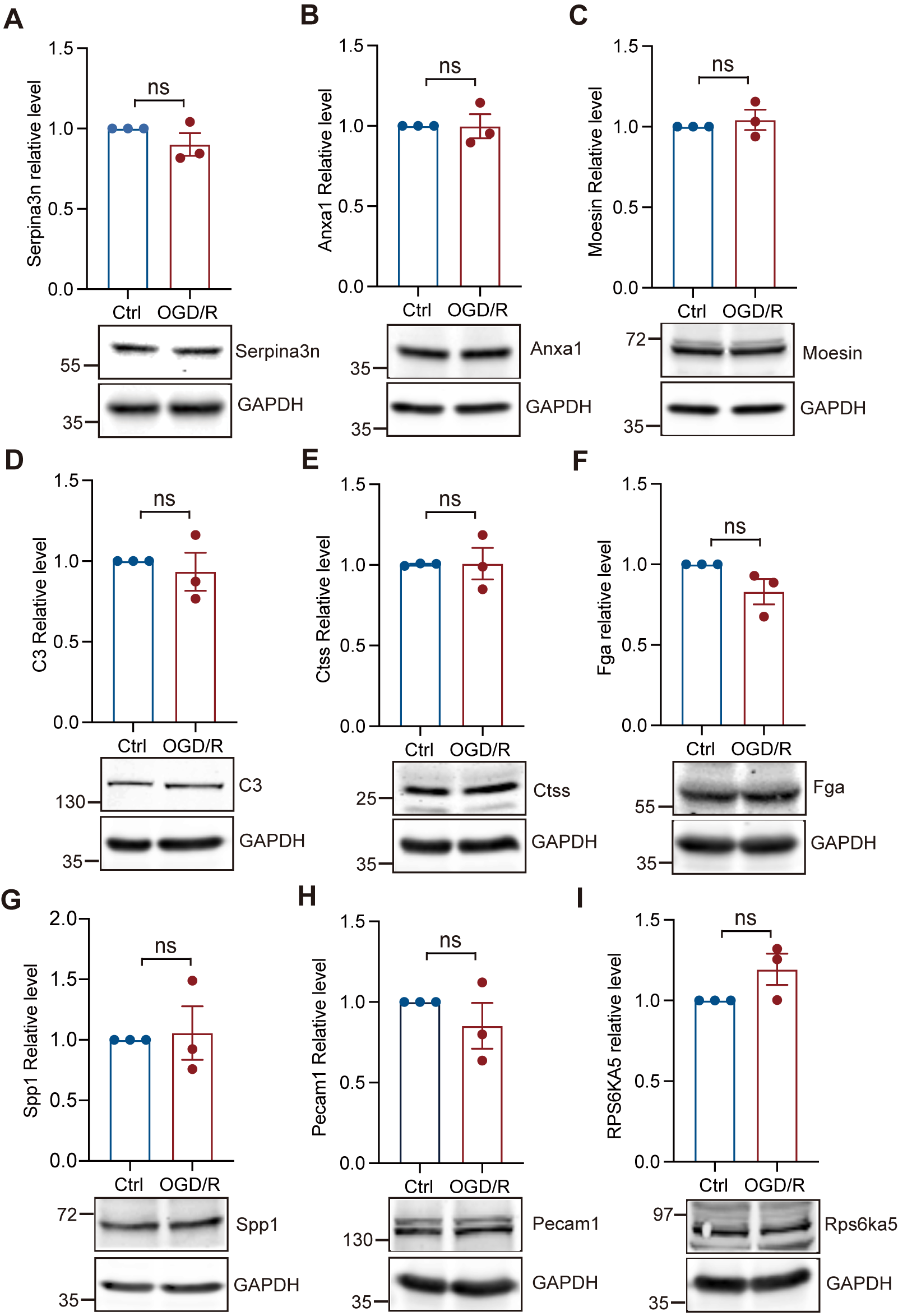


**Supplemental Figure S6. Validation of differentially regulated proteins associated with immune-inflammatory responses in OGD/R-treated BV2 microglial cells. (A-I)** Representative blot images and quantitative analyses of the overlapped immune inflammatory responses related proteins, including Serpina3n, Anxa1, Moesin, C3, Ctss, Fga, Spp1, Pecam1, and Rps6ka5 in BV2 microglia cells with or without OGD/R treatment. Data are shown as mean ± SEM. Statistical significance was determined by unpaired student’s t-test for comparisons between two groups. ns, not significant.


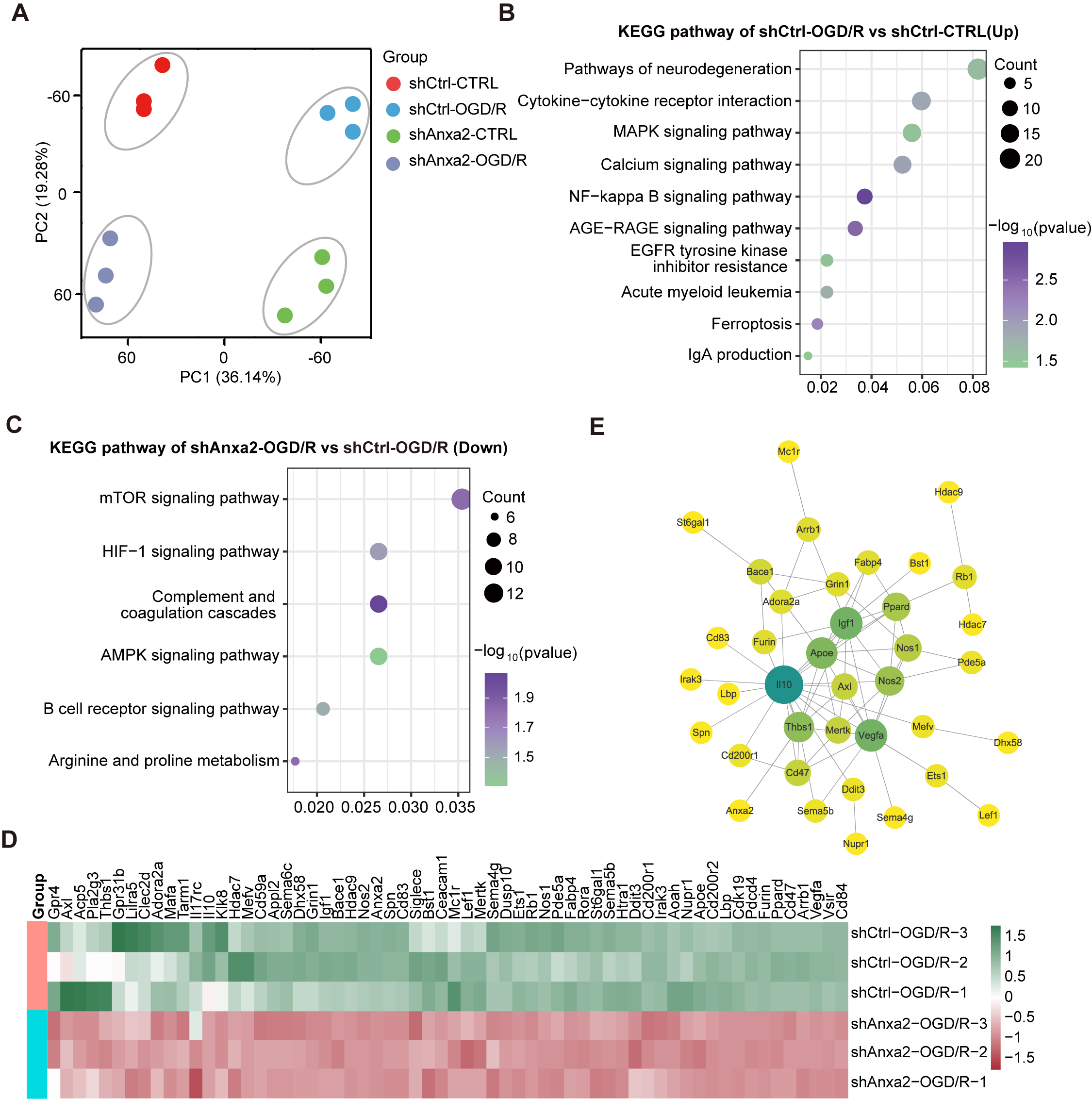


**Supplemental Figure S7. Transcriptome analysis reveals the effect of Anxa2 on inflammatory response after OGD/R in BV2 microglia cells. (A)** PCA analysis for transcriptome data from three biological replicates of shCtrl-CTRL, shCtrl-OGD/R, shAnxa2-CTRL, and shAnxa2-OGD/R samples, respectively. **(B)** KEGG pathway analyses of DEGs up-regulated in shCtrl group after OGD/R (p-value < 0.05). **(C)** KEGG pathway analyses of DEGs down-regulated in shAnxa2-OGD/R versus shCtrl-OGD/R group (p-value < 0.05). **(D)** The PPI network map of differential genes with Anxa2 knockdown after OGD/R. The green nodes represent the hub subnetwork analyzed in cytoscape by cytohubba plug-in. **(E)** Heatmap of DEGs analyzed in BV2 microglial cells with knockdown of Anxa2 after OGD/R using RNA sequencing.


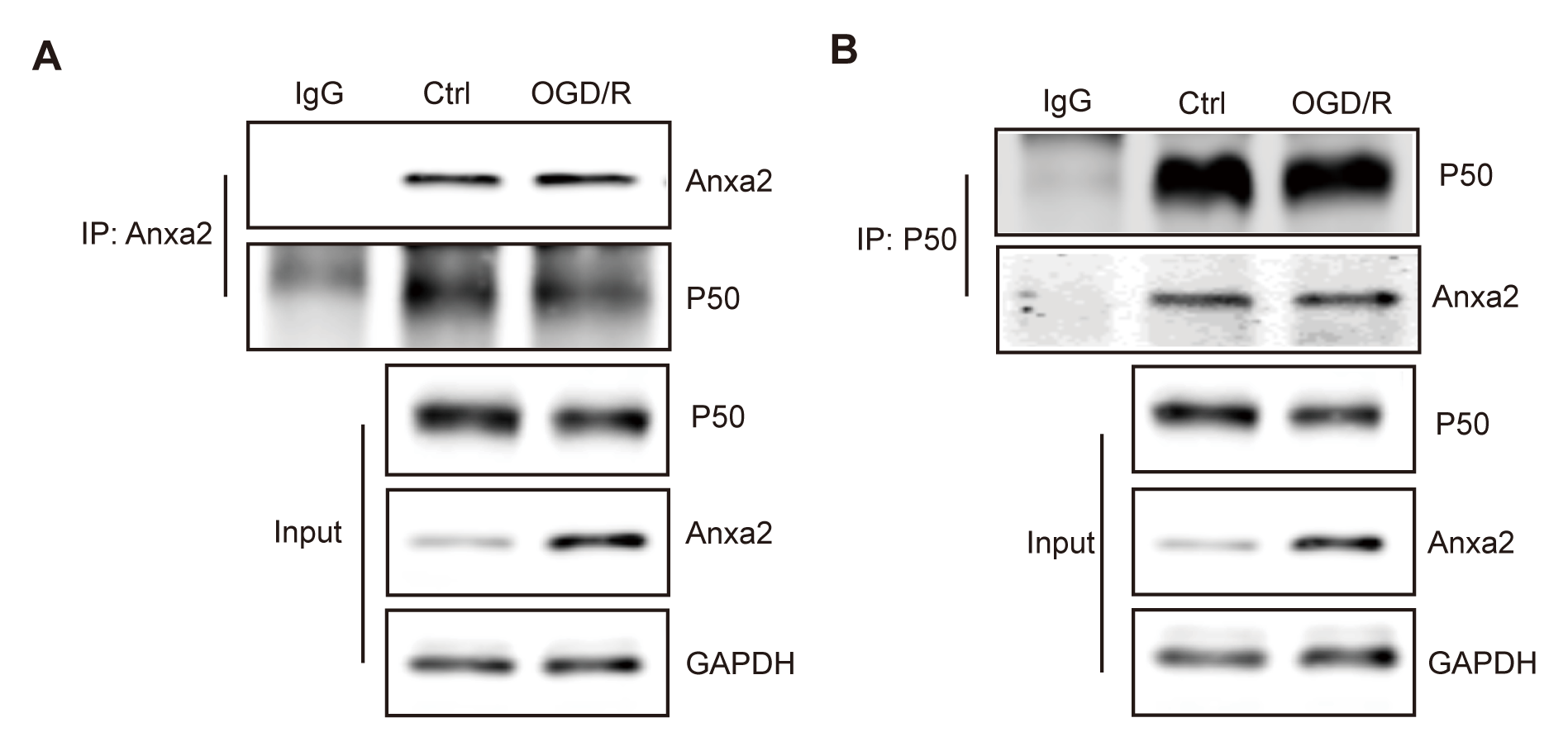


**Supplemental Figure S8. The interaction of Anxa2 with p50 subunit of NF-κB was not affected after OGD/R in BV2 cells. (A and B)** Immunoprecipitation of Anxa2 (A) or P50 (B) with anti-Anxa2 or P50 antibodies followed by Western blot analysis with anti-P50 (A) or anti-Anxa2 (B) antibodies to detect P50 or Anxa2, respectively, in BV2 cells after OGD/R. GAPDH served as loading controls.

Reference

1. Jung, H., Kim, J. S., Kim, W. K., Oh, K. J., Kim, J. M., Lee, H. J., Han, B. S., Kim, D. S., Seo, Y. S., Lee, S. C., Park, S. G., and Bae, K. H. (2015) Intracellular annexin A2 regulates Nf-kB signaling by binding to the p50 subunit: Implications for gemcitabine resistance in pancreatic cancer. *Cell Death and Disease*
